# Discovery of Novel Agents on Spindle Assembly Checkpoint to Sensitize Vinorelbine-Induced Mitotic Cell Death against Human Non-Small Cell Lung Cancers

**DOI:** 10.3390/ijms21165608

**Published:** 2020-08-05

**Authors:** Ya-Ching Chang, Yu-Ling Tseng, Wohn-Jenn Leu, Chi-Min Du, Yi-Huei Jiang, Lih-Ching Hsu, Jui-Ling Hsu, Duen-Ren Hou, Jih-Hwa Guh

**Affiliations:** 1 School of Pharmacy, College of Medicine, National Taiwan University, No. 33, Linsen S. Rd, Taipei 100, Taiwan; r06423014@ntu.edu.tw (Y.-C.C.); d02423201@ntu.edu.tw (W.-J.L.); r07423013@ntu.edu.tw (C.-M.D.); r07423015@ntu.edu.tw (Y.-H.J.); lhsu@ntu.edu.tw (L.-C.H.); 2 Department of Chemistry, National Central University, No. 300 Jhong-Da Road, Jhong-li, Taoyuan 32001, Taiwan; r07223108@ntu.edu.tw; 3 Department of Pharmacy, New Taipei Municipal TuCheng Hospital, Chang Gung Memorial Hospital, New Taipei City 220, Taiwan

**Keywords:** Novel sildenafil mimetic, vinorelbine, spindle assembly checkpoint, apoptotic synergism, BUBR1

## Abstract

Non-small cell lung cancer (NSCLC) accounts about 80% of all lung cancers. More than two-thirds of NSCLC patients have inoperable, locally advanced or metastatic tumors. Non-toxic agents that synergistically potentiate cancer-killing activities of chemotherapeutic drugs are in high demand. YL-9 was a novel and non-cytotoxic compound with the structure related to sildenafil but showing much less activity against phosphodiesterase type 5 (PDE5). NCI-H460, an NSCLC cell line with low PDE5 expression, was used as the cell model. YL-9 synergistically potentiated vinorelbine-induced anti-proliferative and apoptotic effects in NCI-H460 cells. Vinorelbine induced tubulin acetylation and Bub1-related kinase (BUBR1) phosphorylation, a necessary component in spindle assembly checkpoint. These effects, as well as BUBR1 cleavage, were substantially enhanced in co-treatment with YL-9. Several mitotic arrest signals were enhanced under combinatory treatment of vinorelbine and YL-9, including an increase of mitotic spindle abnormalities, increased cyclin B1 expression, B-cell lymphoma 2 (Bcl-2) phosphorylation and increased phosphoproteins. Moreover, YL-9 also displayed synergistic activity in combining with vinorelbine to induce apoptosis in A549 cells which express PDE5. In conclusion. the data suggest that YL-9 is a novel agent that synergistically amplifies vinorelbine-induced NSCLC apoptosis through activation of spindle assembly checkpoint and increased mitotic arrest of the cell cycle. YL-9 shows the potential for further development in combinatory treatment against NSCLC.

## 1. Introduction

Lung carcinoma is one of the top leading causes for cancer death worldwide. Based on histopathological classification, more than 80% of the lung cancer patients are categorized as non-small cell lung cancer (NSCLC) in which the survival rate is lower than 10% for late-stage patients. There are continued unmet needs for more effective therapeutic strategies to better outcome in patients with advanced NSCLC. Drugs that influence microtubule dynamics have been a core in cancer treatment for decades. Vinorelbine, a third-generation *vinca* alkaloid approved for NSCLC treatment by inhibiting microtubule dynamics, either monotherapy or combination with other cancer medications enhances the survival of patients with advanced NSCLC [1,2]. However, treatment toxicities may occur, such as neutropenia, leucopenia, edema, bruising, bleeding, anemia, hair loss, and peripheral neuropathy [3]. Neutropenia is the most common toxicity to discontinue the administration of the medication. Reducing the dose intensity through combination therapy or changing administration schedule is the strategy to improve the tolerability of vinorelbine [1,4]. However, the development of novel drug combination is still needed in improving drug efficacy and increasing tolerability.

Drug repurposing and the development of its derivatives have emerged as an attractive strategy in combating malignant tumors. Phosphodiesterase type 5 (PDE5) inhibitors (e.g., sildenafil and vardenafil) either mono-treatment or combination with cancer therapeutic drugs have been suggested to display anti-proliferative and apoptotic activities *in vitro* and suppress tumor growth *in vivo* against several cancers, such as prostate cancer, lung cancer, breast cancer, colon adenocarcinoma, neuroblastoma, and oral cancer [5,6,7,8,9]. The inhibition of PDE5-dependent signaling has been claimed in decreasing cell migration and sensitizing apoptotic capability of anticancer medications through multiple pathways including an increase of reactive oxygen species (ROS) production, increased CD95-mediated apoptosis, enhanced endocytosis-mediated drug uptake and reduced CXCL16 expression and secretion [5,10,11]. However, PDE5-independent off-target pathways have been reported in sildenafil-mediated apoptotic sensitizing mechanisms [7]. Although the synergistic effects are documented, the potential of PDE5 inhibitors may be dependent on cancer types and chemotherapeutics.

Some reports have demonstrated that using PDE5 inhibitors may be associated with increased risk of melanoma and basal cell carcinoma [12,13,14] although several studies conclude the causal connections need solid evidence between using PDE5 inhibitors and risk of melanoma [15,16]. Dhayade and the colleagues have provided experimental evidence showing a cGMP-dependent growth-promoting signaling in murine and human melanoma cells in which p44/42 mitogen-activated protein kinase and cGMP-dependent protein kinase I also are involved [17]. Moreover, oncogenic BRAF induces invasion through downregulation of PDE5A in melanoma cells [18]. Owing to the potential risk in inducing melanoma and, possibly, orthostatic hypotension in PDE5 inhibitors [19], we have synthesized a series of derivatives structurally mimicking the category of sildenafil while reducing the inhibitory activity of PDE5. To the best of our knowledge, this is the first study in such design to elucidate the combination therapy with vinorelbine against NSCLC. The combination index has been examined based on several anti-proliferative determinations. Furthermore, phosphorylation of Bub1-related kinase (BUBR1, a key component of mitotic checkpoint for spindle assembly) [20], tubulin acetylation and cell cycle regulator proteins have been identified in the study to discover the sensitization mechanism in which the potential derivative drives.

## 2. Results

### 2.1. YL-9 Shows Less Anti-PDE5 Activity But Reserves Sensitizing Capability to Vinorelbine-Induced Anti-NSCLC Effect

**Figure 1:**
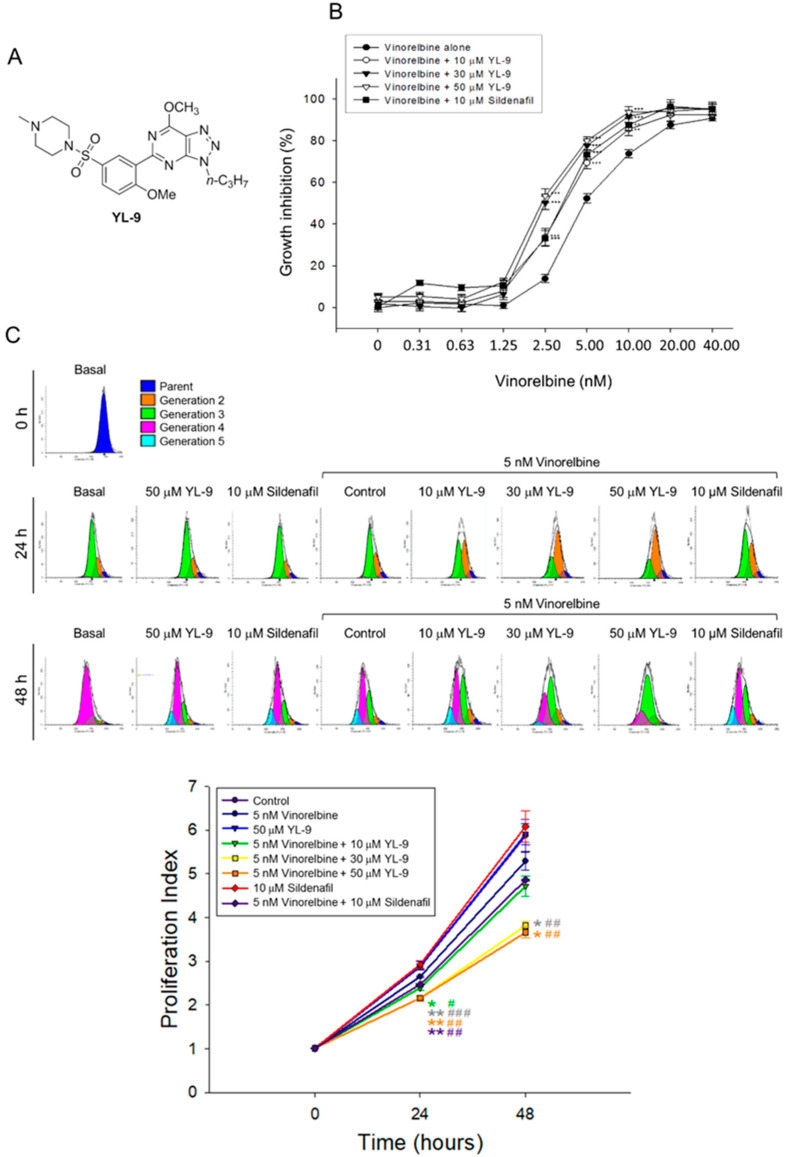
Effect of YL-9 on vinorelbine-induced anti-proliferation in NCI-H460 cells. (**A**) Chemical structure of YL-9. (**B**) The cells were incubated with various concentrations of vinorelbine in the absence or presence of YL-9 or sildenafil for 48 h. After the treatment, the cell proliferative effect was determined using sulforhodamine B (SRB) assay. Data are expressed as mean ± SEM of six independent experiments. ** *p* < 0.01 and *** *p* < 0.001 compared with vinorelbine alone. (**C**) The cells were incubated with the indicated agent for 24 or 48 h. After the treatment, the cell proliferative effect was determined using flow cytometric analysis of carboxyfluorescein succinimidyl ester (CFSE) staining and the proliferation index was analyzed with ModFit LT^TM^ 3.3 (Verity Software House, Topsham, ME, USA). Data are expressed as mean ± SEM of three independent experiments. * *p* < 0.05 and ** *p* < 0.01 compared with the control. ^#^ *p* < 0.05, ^##^ *p* < 0.01 and ^###^ *p* < 0.001 compared with vinorelbine alone.

The combination effect between vinorelbine and YL-9 (structure, Figure 1A) on cell proliferation in NCI-H460 cells was determined. NCI-H460 is a good cell model in studying anti-NSCLC research as the cells may harbor stem-like cells in readily forming anchorage-independent floating spheres, displaying high proliferative potential [21]. Furthermore, NCI-H460 is the cell line showing the least expression of PDE5 among various NSCLC cell lines [22] and enables the study of PDE5-independent signaling pathways in the present work. YL-9 exhibited similar potency to sildenafil in sensitizing vinorelbine-induced inhibition of cell proliferation in NCI-H460 cells using sulforhodamine B (SRB) assay. The IC_50_ of vinorelbine alone was 5.16 ± 0.31 nM. It was significantly shifted to 3.41 ± 0.25 and 2.57 ± 0.13 nM in the presence of YL-9 at 10 and 30 μM, respectively (Figure 1B). The synergism between vinorelbine and YL-9 was assessed through constructing isobolograms and calculating combination index (CI) values using Chou-Talalay method [23]. The resulting CI values were less than 1.0 confirming the synergistic effects (Table 1). Flow cytometric analysis of carboxyfluorescein succinimidyl ester (CFSE) staining, which monitored distinct generations of proliferating cells by fluorescence dye dilution, was used to further examine cell proliferation. The data demonstrated that the proliferation index was significantly decreased in the combinatory exposure to vinorelbine and YL-9 at both 24 and 48 h (Figure 1C). Moreover, the long-term effect (8 days) on anchorage-dependent growth of NCI-H460 cells was examined using clonogenic assay, showing that YL-9 significantly shifted the IC_50_ of 2.22 ± 0.12 nM at vinorelbine alone to 1.19 ± 0.15 (*p* < 0.01) and 0.92 ± 0.05 nM (*p* < 0.001) in the presence of 10 and 30 μM YL-9, respectively (Figure S1). Altogether, the data verified the synergism between vinorelbine and YL-9 on the anti-proliferative effect. Notably, YL-9 was far less effective than sildenafil in inhibiting PDE5A1 activity (IC_50_, 632.2 *v.s.* 3.8 nM) using quantitative enzyme activity assay. The data indicated that the anti-proliferative sensitization was not closely related to the inhibitory effect of PDE5A1 activity.

### 2.2. YL-9 Sensitizes Vinorelbine on Inducing Caspase Activation and Apoptosis in NCI-H460 Cells

**Figure 2:**
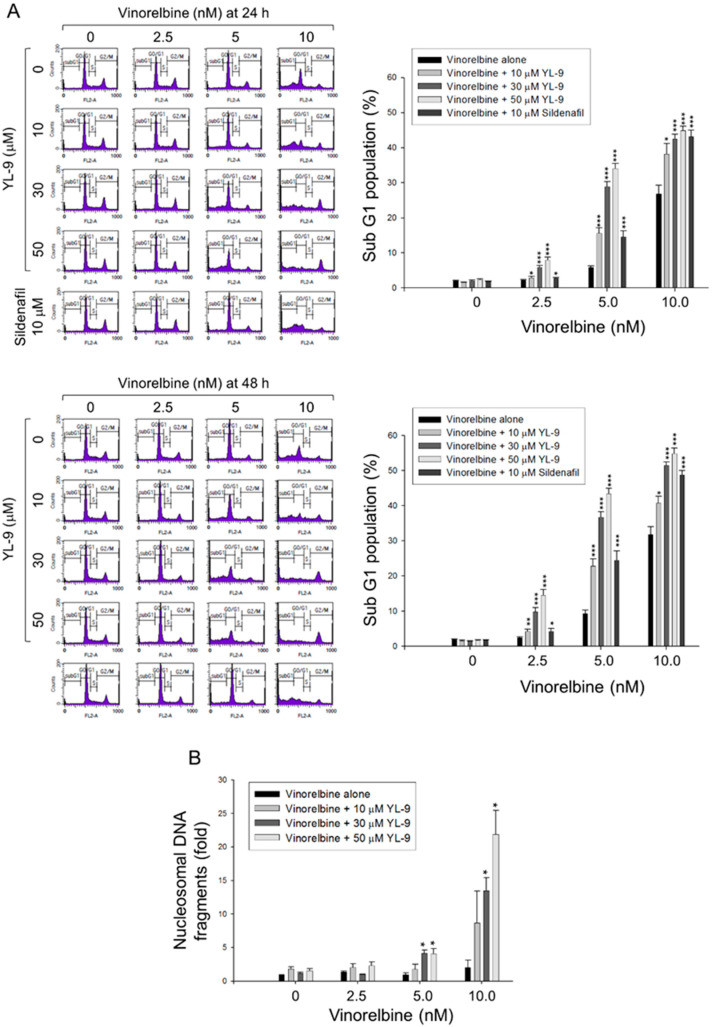
Effect of YL-9 on vinorelbine-induced apoptosis in NCI-H460 cells. (**A**) The cells were incubated with various concentrations of vinorelbine in the absence or presence of YL-9 or sildenafil for 24 or 48 h. After the treatment, the cells were harvested for the detection of cell populations at different cell cycle phases using flow cytometric analysis of propidium iodide staining. The sub-G1 populations (apoptosis) were presented as mean ±SEM of six independent experiments. (**B**) The cells were incubated with the indicated agents for 24 h. Cell apoptosis was examined by detecting nucleosomal DNA fragmentation using Cell Death Detection ELISA^PLUS^ kit. Data are expressed as mean ± SEM of three independent experiments. * *p* < 0.05, ** *p* < 0.01 and *** *p* < 0.001 compared with vinorelbine alone.

Cell apoptosis was quantitatively examined using flow cytometric analysis of PI staining that detected fragmented DNA in sub-G1 population. The data showed that YL-9 resulted in a concentration-dependent synergistic effect on vinorelbine-induced increase of sub-G1 population at both 24- and 48-h treatments (Figure 2A). Furthermore, YL-9 displayed a concentration-dependent potentiation of vinorelbine-induced increase of nucleosomal DNA fragmentation based on the quantitation of cytoplasmic histone-associated DNA fragments in cells. (Figure 2B). The data suggested the synergistic effect between vinorelbine and YL-9 on inducing cell apoptosis.

Caspase-9 is the most intensively studied initiator caspase that plays a key role in mitochondria-involved intrinsic apoptosis pathway. In contrast, both caspase-3 and -7 are known for their critical role in the execution of apoptosis. The exposure of NCI-H460 cells to 5 nM vinorelbine for 24 h induced a negligible increase of caspase activation, whereas the combination of vinorelbine and YL-9 resulted in a remarkable caspase activation, including caspase-9, -3, and -7 (Figure S2).

### 2.3. YL-9 Synergistically Potentiates Vinorelbine-Induced Mitotic Spindle Defects and Mitotic Arrest of the Cell Cycle

**Figure 3:**
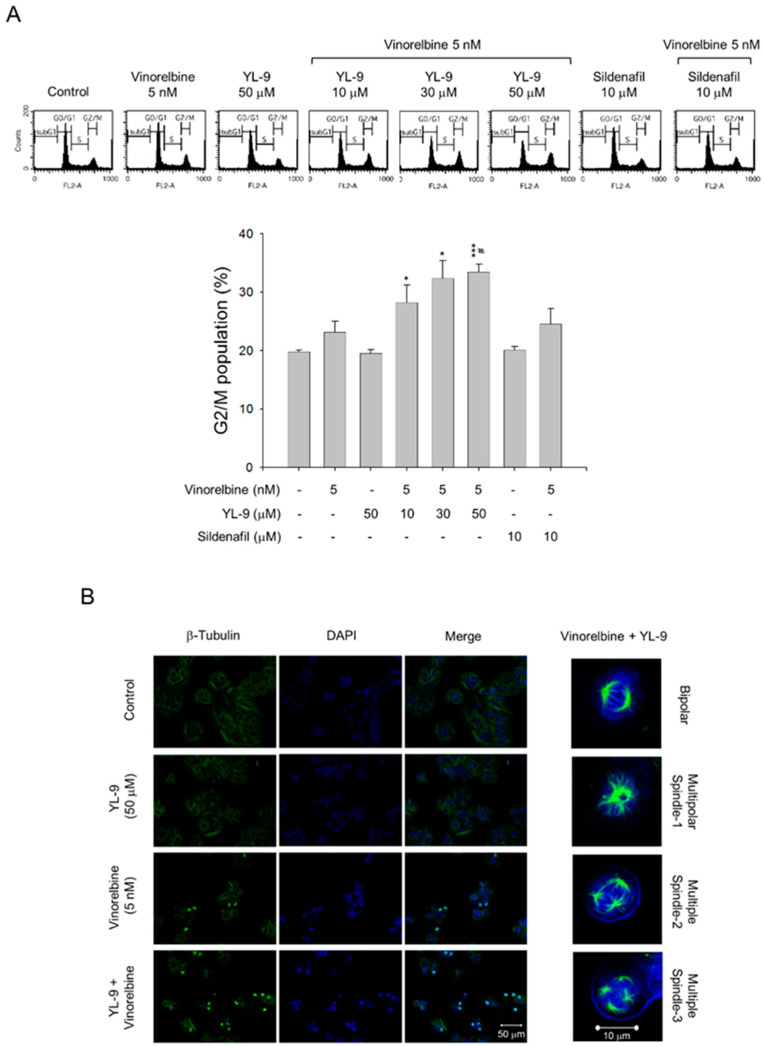
Effect of YL-9 on vinorelbine-induced accumulation of G2/M population and mitotic spindle abnormalities. (**A**) NCI-H460 cells were incubated with the indicated agents for 6 h. After the treatment, the cells were harvested for the detection of cell populations at different cell cycle phases using flow cytometric analysis of propidium iodide staining. The G2/M populations were presented as mean ± SEM of three independent experiments. * *p* < 0.05 and *** *p* < 0.001 compared with the control. ^#^ *p* < 0.05 compared with vinorelbine alone. (**B**) The cells were incubated with the indicated agents for 24 h. After the treatment, the cells were fixed with methanol and stained with β-tubulin antibody (green, microtubules) and DAPI (blue, nucleus). The mitotic spindle organization was detected by immunofluorescence confocal microscopic assay. The organizations of mitotic spindles, including bipolar and multipolar, induced by the combinatory exposure to vinorelbine and YL-9 were demonstrated.

*Vinca* alkaloids are a class of microtubule inhibitors binding to the vinca domain on β-tubulin that at clinically relevant doses (e.g., low nanomolar) can kinetically stabilize microtubules, leading to mitotic accumulation at metaphase-anaphase transition, chromosome missegregation, mitotic catastrophe and apoptosis [24]. The data demonstrated that the cell population at G2/M phase was 39.47 ± 0.86% at 5 nM vinorelbine and was markedly increased to 44.36 ± 2.06%, 48.11 ± 1.72%, and 49.54 ± 1.28% in the presence of YL-9 at 10, 30, and 50 μM, respectively (Figure 3A). Confocal microscopic examination using fluorescence staining at β-tubulin and chromosome showed that vinorelbine alone induced an increase of mitotic spindle abnormalities (46.44 ± 8.43% *v.s.* control of 3.43 ± 1.92%, *p* < 0.01). The effects were dramatically sensitized in the simultaneous presence of YL-9 (85.70 ± 4.27%, *p* < 0.001 *v.s.* control and *p* = 0.01 *v.s.* vinorelbine alone), showing abnormal bipolar and multipolar spindles with misaligned chromosome around the spindles (Figure 3B).

### 2.4. YL-9 Potentiates Vinorelbine-Induced Phosphorylation and Cleavage of BUBR1

**Figure 4:**
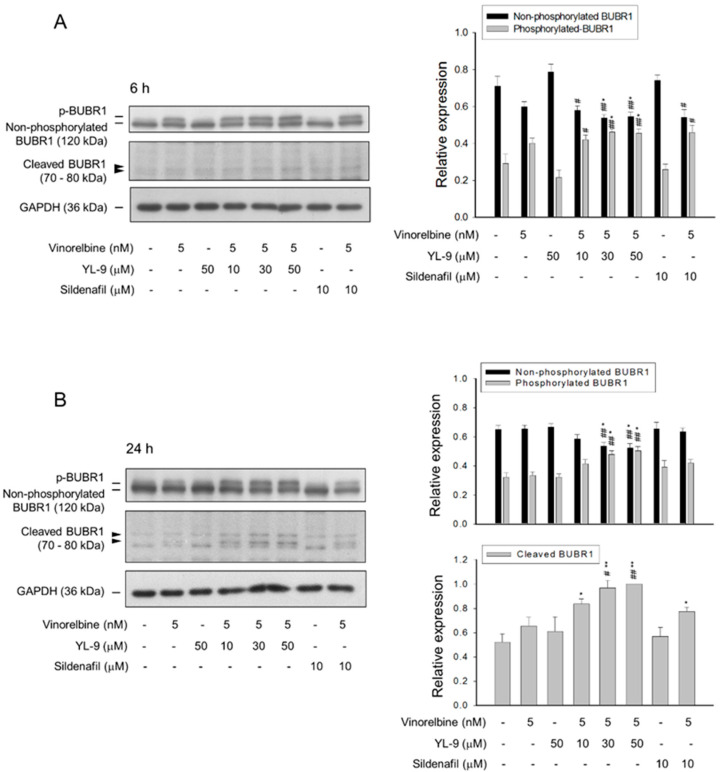
Effect of YL-9 on vinorelbine-induced phosphorylation and cleavage of BUBR1. NCI-H460 cells were incubated with the indicated agents for 6 (**A**) or 24 h (**B**). After the treatment, the cells were harvested for the detection of protein expression using Western blotting analysis. The protein expressions of total BUBR1, including non-phosphorylated and phosphorylated BUBR1 were quantified using Bio-Rad Image Lab^TM^ Software. The expressions of total BUBR1 in each treatment group are summed up as 1.0 of the relative expression (*y*-axis). The quantitative data of expressions in cleaved BUBR1, which account for minor portion of BUBR1 expressions, at a 24-h treatment are shown separately and the expression is relative to the group of 5 nM vinorelbine/50 μM YL-9. Data are expressed as mean ± SEM of three independent experiments. * *p* < 0.05 and ** *p* < 0.01 compared with the control. ^#^ *p* < 0.05 and ^##^ *p* < 0.01 compared with vinorelbine alone.

BUBR1, a necessary component of the spindle assembly checkpoint (SAC), plays a critical role in kinetochore localization of various spindle checkpoint proteins in mitosis [20]. Our data showed that YL-9 synergistically potentiated vinorelbine on inducing the formation of aberrant spindle formation. Therefore, the effect on BUBR1 was determined. The data demonstrated that vinorelbine induced an increase of BUBR1 phosphorylation which was markedly enhanced at a 6-h co-treatment with YL-9 (Figure 4A). Notably, a 24-h co-treatment of the cells with vinorelbine and YL-9 resulted in a loss of BUBR1 protein levels associated with a significant increase of the cleaved forms of BUBR1 (Figure 4B). BUBR1 phosphorylation is suggested to enhance the chromosome alignment and mitotic checkpoint [25]. The depletion of BUBR1 causes the misalignment of chromosome and aneuploidy, leading to an increase of cell apoptosis [26]. The data suggested that YL-9 increased vinorelbine-mediated BUBR1 phosphorylation and, in particular, BUBR1 cleavage which were responsible for the synergistic increase of misaligned chromosome and mitotic spindle defects.

### 2.5. YL-9 Sensitizes Vinorelbine-Induced Increase of Mitotic Protein Expression and Phosphorylation

**Figure 5:**
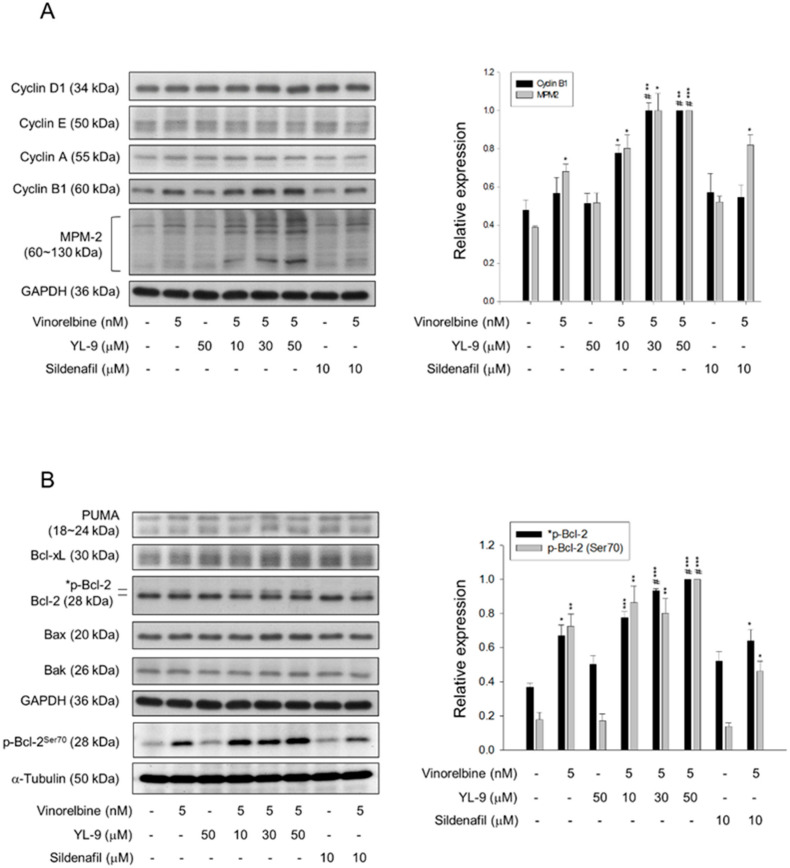
Effect of YL-9 on vinorelbine-induced alteration of several protein expressions. NCI-H460 cells were incubated with the indicated agents for 6 or 24 h. After the treatment, the cells were harvested for the detection of the expressions of cyclin proteins and phosphorylated mitotic protein (MPM-2) (**A**) or Bcl-2 family members (**B**) using Western blotting analysis. The expression was quantified using Bio-Rad Image Lab^TM^ Software. The expression is relative to the group of 5 nM vinorelbine/50 μM YL-9. The images of internal control GAPDH are re-used in Figure 5A,B because they are the same experiment. Data are expressed as mean ± SEM of three independent experiments. * *p* < 0.05, ** *p* < 0.01 and *** *p* < 0.001 compared with the control. ^#^ *p* < 0.05 compared with vinorelbine alone.

Since various cyclin proteins and associated Cdk activities are involved in cell cycle control, the expression levels of cyclin proteins were determined. Cyclin B1/Cdk1 complex activity plays a key role in mitosis. Our data showed that although not significant, vinorelbine induced a moderate increase of cyclin B1 protein expression. YL-9 synergistically increased vinorelbine-induced effect on cyclin B1 expression other than that on cyclin D1, cyclin E and cyclin A (Figure 5A). Furthermore, YL-9 concentration-dependently sensitized vinorelbine in inducing a dramatic increase of phosphorylated mitotic proteins (Figure 5A). In parallel experiments, several pro-apoptotic and anti-apoptotic B-cell lymphoma 2 (Bcl-2) family proteins were examined. YL-9, by itself and in combination with vinorelbine, showed insignificant effect on the protein expression of several Bcl-2 derivatives, including p53 upregulated modulator of apoptosis (PUMA), Bax, Bak, and Bcl-xL. In contrast, YL-9 enhanced vinorelbine-induced Bcl-2 phosphorylation (Figure 5B). Phosphorylated Bcl-2 has been evident to be present in mitosis simultaneously in peak cyclin B1/Cdk1 complex activity. Moreover, Bcl-2 phosphorylation has a critical role in the control of mitotic death under the exposure to microtubule inhibiting agents [27]. Our data supported that YL-9 synergistically potentiated vinorelbine in triggering mitotic arrest of the cell cycle and mitotic death. Notably, Bak activation has been suggested by the conversion from a latent to an active form that involves an exposure of an occluded N-terminal epitope of Bak [28]. Flow cytometric analysis was performed to examine the exposed N terminus of Bak. The data showed that the combinatory treatment with vinorelbine and YL-9 induced a small but significant increase in Bak activity (Figure S3), suggesting the involvement of active Bak in apoptotic response.

### 2.6. YL-9 Potentiates Vinorelbine-Induced Tubulin Acetylation on Lys40

**Figure 6:**
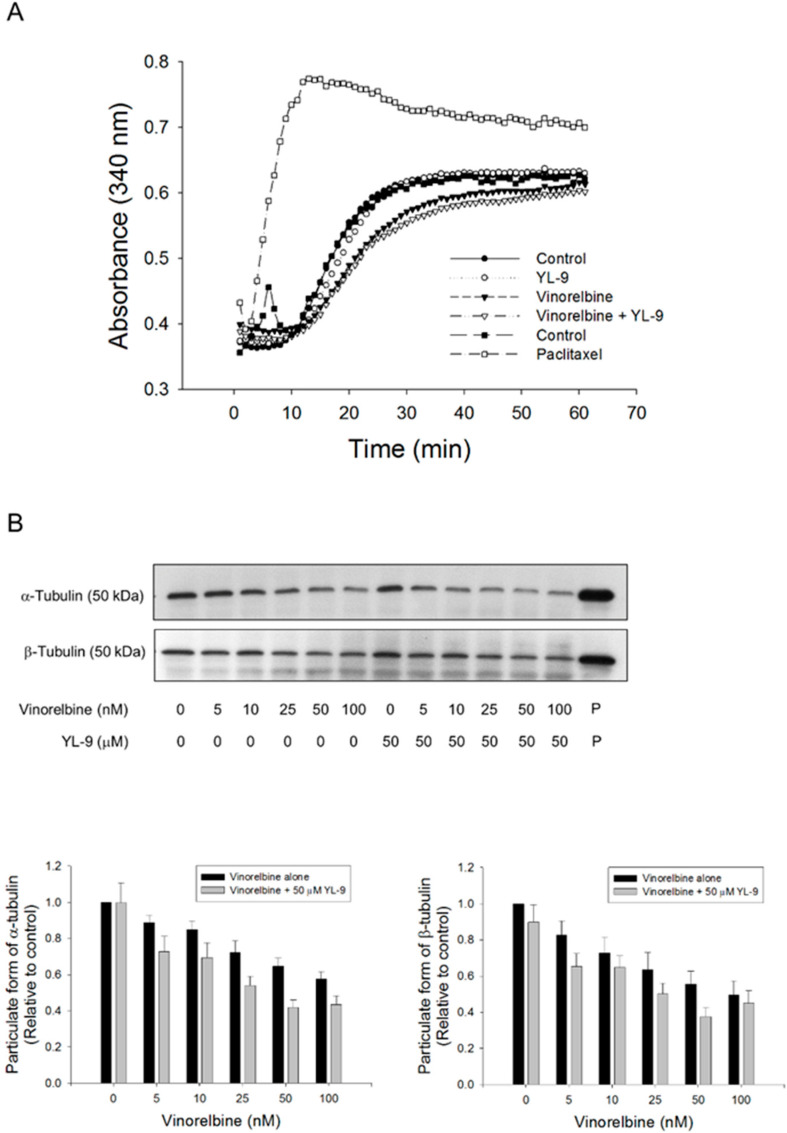
Effect of YL-9 on vinorelbine-induced alteration of tubulin polymerization. (**A**) Tubulins were incubated in the absence or presence of YL-9 (50 μM) or vinorelbine (1 μM) at 37 °C for 1 h. The levels of tubulin polymerization were detected using ELISA reader at 340 nm absorbance. Positive control, 30 μM paclitaxel. (**B**) NCI-H460 cells were incubated in the absence or presence of YL-9 or vinorelbine for 6 h. The cells were harvested for the detection of tubulin polymerization (particulate form of tubulin) using Western blotting analysis. The expression was quantified using Bio-Rad Image Lab^TM^ Software. The expression is relative to compound-free control. Data are expressed as mean ± SEM of eight independent experiments. P, paclitaxel as positive control.

Mitotic spindle comprises microtubules of polar dynamic fibers that polymerize from tubulin subunits and a variety of other proteins functioning mutually to control chromosome segregation [29]. The tubulin polymerization was examined using both *in vitro* pure tubulin assay model and in cell microtubule assembly assay in this study. Because there are no related enzymes and proteins but only pure tubulins in the *in vitro* assay system, high concentrations of vinorelbine (1000 nM) was used that caused moderate inhibition (16.7%) of tubulin polymerization. YL-9 neither modified the basal levels nor synergized vinorelbine-induced inhibitory effect (Figure 6A). In contrast, the cell model showed that vinorelbine concentration-dependently inhibited, while the reference agent paclitaxel induced, the formation of polymerized microtubule (particulate form). Although not significant, YL-9 caused a small increase of vinorelbine-mediated effect (Figure 6B).

**Figure 7:**
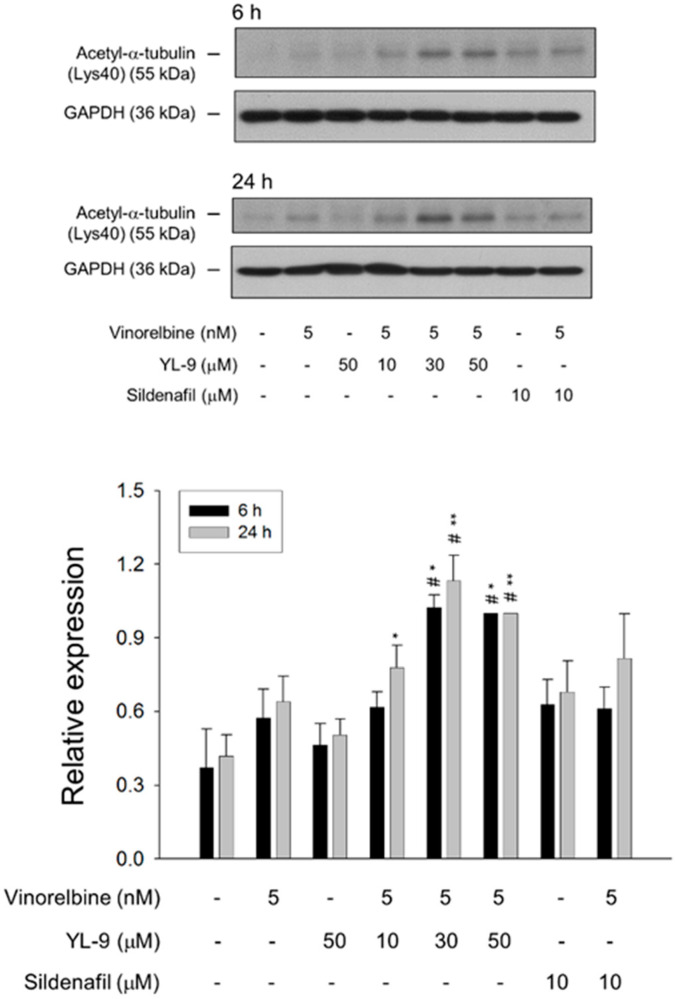
Effect of YL-9 on vinorelbine-induced acetylation of α-tubulin. NCI-H460 cells were incubated with the indicated agents for 6 or 24 h. After the treatment, the cells were harvested for the detection of protein expression using Western blotting analysis. The expression was quantified using Bio-Rad Image Lab^TM^ Software. The expression is relative to the group of 5 nM vinorelbine/50 μM YL-9. Data are expressed as mean ± SEM of three independent experiments. * *p* < 0.05 and ** *p* < 0.01 compared with the control. ^#^ *p* < 0.05 compared with vinorelbine alone.

Tubulin is subject to various post-translational modifications in which tubulin acetylation is considered to be a marker of microtubule stabilization [30,31]. The level of tubulin acetylation was examined using Western blot analysis in NCI-H460 cells. Only a few microtubules were acetylated in the control cells, whereas vinorelbine at a low concentration (5 nM) induced an increase of tubulin acetylation on Lys40. The effect was significantly increased in the presence of YL-9 (Figure 7).

## 3. Discussion

Vinorelbine as a single agent or in combination regimens is clinically used for the treatment of NSCLC. The study was based on furthering drug repurposing to new chemical entities that show less side effects while synergistically potentiate vinorelbine-induced anti-NSCLC activity. YL-9 was designed and developed based on these rationales. The stability of YL-9 in the medium was examined to be at least for 48 h (Figure S4). YL-9, by itself, did not affect the cell proliferation (Figure S1), survival (Figure 2 and Figure S2) and cell cycle progression (Figure 3A). The sensitization activity of YL-9 on vinorelbine-induced effect was independent of the suppression of PDE5 because the experiments were performed in NCI-H460, a cell line with low PDE5 expression [22]. Furthermore, YL-9 displayed equal sensitization activity to sildenafil in vinorelbine-induced effects, although its anti-PDE5 activity was 167 times less than that of sildenafil. YL-9, which is designed to retain the synergistic effect on vinorelbine-induced killing activity in NSCLC, may have decreased risk in causing the adverse effects, including melanoma, basal cell carcinoma, and orthostatic hypotension, which may be induced in patients who use PDE5 inhibitors [12,13,14,19]. Notably, YL-9 also displayed synergistic activity in combining with vinorelbine to induce apoptosis in A549 cells which express PDE5 (Figure S5).

Dynamic instability, the switching between growing and shortening of microtubules, is critical for the chromosomes to equatorial metaphase plate and tension-guided oscillations of the chromosomes at both prometaphase and metaphase [32,33]. *Vinca* alkaloids induce microtubule depolymerization and suppress treadmilling and dynamic instability, leading to mitotic arrest of the cell cycle and the inhibition of cell proliferation [33]. Our data showed that YL-9 induced an insignificant increase of vinorelbine-inhibited formation of polymerized microtubule. Furthermore, YL-9 induced a profound synergistic increase of vinorelbine-mediated cell population arresting at mitotic phase and apoptotic sensitization. The data suggest a possible impact of YL-9 on vinorelbine-suppressed microtubule dynamic instability. Recently, whether the relationship between microtubule stabilization and acetylation of Lys40 in α-tubulin is correlative or causative remains a point of contention. Eshun-Wilson and colleagues performed the assay using pure samples of α-tubulin acetyltransferase αTAT1-acetylated and SIRT2-deacetylated microtubules by means of high-resolution cryo-electron microscopic examination. They reported that the acetylation causes conformational change of the flexible loop which contains Lys40 acetylation. It decreases the disorder of the loop and constrains the states of the sampling, suggesting that the acetylation of α-tubulin can affect microtubule stability [34]. The study supports the present data that the increased Lys40 acetylation of α-tubulin in the presence of vinorelbine and YL-9 may possibly contribute to the stabilization of microtubule dynamics.

A profound effect under combinatory treatment was the formation of multipolar spindle abnormalities surrounded with misaligned chromosome. A normal bipolar spindle is the cell undergoing centrosome duplication during mitosis with strictly controlled processes [35]. A defect in the process leads to multipolar spindle abnormalities and flaws in chromosome distribution. Multiple centrosomes can be generated by centrosome amplification or centrosome splitting into multiple microtubule organizing centers. Intervention in the functions of spindle related proteins and premature splitting of centrosomal materials can also contribute to multipolar spindle abnormalities [36,37,38]. Confocal microscopic examination using fluorescence staining at tubulin and chromosome demonstrated that YL-9 dramatically synergized vinorelbine in inducing abnormal bipolar and multipolar spindles with misaligned chromosome around the spindles. These effects resulted in significant sensitization of cell apoptosis. Of note, a 10-time apoptotic potentiation was observed at non-toxic concentration (50 μM) of YL-9 using nucleosomal DNA fragmentation assay (Figure 2B).

SAC is a surveillance mechanism functioning at the mitotic phase of cell cycle to ensure accurate segregation of chromosomes by delaying the onset of anaphase until all chromosomes are appropriately bi-oriented on mitotic spindle. Disrupting microtubule dynamics with microtubule inhibiting agents activates the SAC and induces mitotic cell death. Notably, mitotic slippage may occur prematurely without accurate chromosomal segregation or cytokinesis [39]. The rate of mitotic slippage is positively correlated to the rate of cyclin B1 ubiquitination and degradation in active SAC condition [40,41]. Numerous studies have documented that mitotic slippage can limit the effectiveness of microtubule poisons [42]. Besides, such agents may induce high numbers of genetically abnormal cells if mitotic slippage occurs at a high rate [40]. Our data show that the combinatory treatment with vinorelbine and YL-9 dramatically enhances and keeps the high levels of cyclin B1 protein, which are believed to retard the mitotic slippage during the SAC activation. These effects are, at least partly, responsible to the facilitated cell death in this study. BUBR1, a multi domain protein kinase, is one of the key components of the SAC functioning as a mitosis-safeguard protein to ensure perfect order of mitotic progression. BUBR1 concentrates on kinetochores during prophase and more BUBR1 proteins are connected to kinetochores of unaligned chromosomes [43,44]. BUBR1 phosphorylation appears to be a key mechanism in controlling BUBR1 activity during SAC [44]. Further studies have shown that BUBR1 is phosphorylated on several residues, including Ser435, Ser543, Ser670, Ser676, and Ser1043, which are dependent on the activities of aurora B, polo-like kinase 1 or monopolar spindle 1 kinases [45,46,47]. Our data demonstrated that YL-9 profoundly synergized with vinorelbine on inducing BUBR1 phosphorylation, suggesting increased SAC activation and spindle disruption. Furthermore, the combinatory treatment caused the cleavage of BUBR1. It was suggested to be attributed to the activation of caspase-3 since BUBR1 was identified to be cleaved by caspase-3 at two sites: primarily at Asp607/Asp610 and secondarily at Asp576/Asp579 [48]. Because cancer cells with flawed checkpoint proteins may be more vulnerable than normal cells to microtubule poisons [33], the cleavage and decrease of BUBR1 protein under the combinatory treatment with vinorelbine and YL-9 could further facilitate the cells into cell death.

Bcl-2, Bcl-xL, and Mcl-1 are three pro-survival Bcl-2 family members that are widely identified to be phosphorylated under the stress of microtubule inhibiting agents. However, their role in mitotic death remained elusive. Recently, Eichhorn and the colleagues examined the individual role of these pro-survival proteins and their phosphorylated forms in mitotic death in HeLa cells using siRNA knockdown of Cdc20, an anaphase-promoting complex activator [27]. Cdc20 knockdown enhanced phosphorylation of Bcl-2, Bcl-xL, and Mcl-1 that were stably overexpressed in the cells. Bcl-2 and Bcl-xL overexpression inhibited Cdc20 knockdown-mediated mitotic death. Phospho-defective mutants showed higher protective effect than wild-type proteins. In contrast, phospho-mimic Bcl-xL failed to protect the cells from mitotic death. Notably, Mcl-1 overexpression also failed to rescue the Cdc20-knockdown cells since it was prone to degradation [27]. These results support that phosphorylation of anti-apoptotic Bcl-2 proteins displays a crucial role in regulating mitotic death. Our data demonstrated that YL-9 induced a profound increase of vinorelbine-mediated Bcl-2 phosphorylation in NCI-H460 cells and were consistent with the suggestion that Bcl-2 phosphorylation was considered the priming signaling in mitotic death pathway.

In conclusion, it is suggested that YL-9 is a novel compound that bypasses the inhibition of PDE5 but enforces the sensitization activity on vinorelbine-induced mitotic cell death through multiple synergistic effects. YL-9 dramatically enhances vinorelbine-induced tubulin acetylation and BUBR1 phosphorylation. Mitotic spindle abnormalities are synergistically increased to form abnormal bipolar and multipolar spindles with misaligned chromosome, thus promoting the mitotic arrest and rendering the cells to mitochondrial dysfunctions. Bcl-2 phosphorylation may contribute to priming the mitochondrial stress and activation of caspase cascades that further cleave BUBR1 to exacerbate misaligned chromosome and mitotic spindle defects and, ultimately, cell death.

## 4. Materials and Methods

### 4.1. Materials

Human NSCLC cell line NCI-H460 was from American Type Culture Collection (Rockville, MD, USA). RPMI 1640 medium, fetal bovine serum (FBS), Pen-Strep-Ampho solution (unit/mL penicillin, 10 mg/mL streptomycin and 0.025 mg/mL amphotericin B) were from GIBCO/BRL Life Technologies (Grand, Island, NY, USA). Vinorelbine, propidium iodide (PI), sulforhodamine B (SRB), Triton X-100, dithiothreitol, phenylmethylsulfonyl fluorid (PMSF) and monoclonal antibody of β-tubulin were from Sigma-Aldrich (St. Louis, MO, USA). Carboxyfluorescein succinimidyl ester (CFSE) was from Thermo Fisher Scientific (Waltham, MA, USA). The following monoclonal antibodies were used: α-tubulin (cat#sc-5286), Bcl-xL (cat#sc-8392), Bcl-2 (cat#sc-7382), Bax (cat# sc-23959), Bak (cat#sc-832), cyclin E (cat# sc-247), cyclin A (cat# sc-596), cyclin B1 (cat#sc-594), GAPDH (cat#sc-32233), PARP-1 (cat#sc-7150), PUMA (cat#sc-28226) and horseradish peroxidase (HRP)-conjugated anti-mouse and anti-rabbit IgGs (Santa Cruz Biotechnology, Santa Cruz, CA, USA), acetyl-α-tubulin (cat#5335), p-Bcl-2 (cat#2827), cleaved caspase-9 (cat9501), caspase -7 (cat#9492) (Cell Signaling, Beverly, MA, USA), cyclin D1 (cat#ab134175), BUBR1 (cat#ab54894) (ABCam, Cambridge, MA, USA), caspase-3 (cat#610322) (Imgenex, San Diego, CA, USA), MPM-2 (cat#05–368), and anti-Bak antibody (N terminus, cat#AM03) (Millipore, Burlington, MA, USA). Bio-Rad protein assay kit was purchased from Bio-Rad (Hercules, CA, USA). Polyvinylidene fluoride (PVDF) membrane was from Pall Gelman Laboratory (Ann Arbor, MI, USA). YL-9 was designed and synthesized by Duen-Ren Hou and Yu-Ling Tseng. The synthesis detail and NMR spectroscopy analysis were provided in Supplementary Materials. The purity of YL-9 is more than 97% by high performance liquid chromatography (HPLC).

### 4.2. Cell Culture

NCI-H460 cells were cultured in RPMI 1640 medium with 10% (*v/v*) heat-inactivated FBS, 4500 mg/L glucose, 100 unit/mL penicillin, and 100 μg/mL streptomycin and were maintained in a 37 °C incubator with 5% CO_2_.

### 4.3. PDE5A1 Activity Assay

The PDE5 enzyme activity was determined using PDE5A1 assay kit (BPS Bioscience, San Diego, CA, USA) based on the PDE5 catalytic ability to hydrolyze dye-labeled cyclic monophosphates, which can further be bound by selective beads and lead to slower rotating and lower polarized light emission. The compounds that inhibited PDE5 enzyme demonstrated affected polarized fluorescence. Following the manufacturer’s protocol, after incubating compounds with PDE5A1 enzyme and FAM-Cyclic-3′, 5′-GMP in buffers for 1 h, the fluorescent polarization was detected and calculated.

### 4.4. SRB and Clonogenic Assays

The cells were cultured in 96-well plates in RPMI medium with 10% FBS at a density of 4000 cells per well for 24. Some cells were then fixed with 10% trichloroacetic acid (TCA) representing cell numbers at the time of drug treatment (T_Z_). Cells in control group were treated with 0.1% dimethyl sulfoxide (DMSO), while cells in experiment group were treated with indicated compounds for 48 h. After treatment, the cells were fixed with 10% TCA and stained with 0.4% (*w/v*) SRB dissolved in 1% acetic acid. Unbound dye was washed out with 1% acetic acid, and SRB bounded cells were solubilized with 10 mM Tris base. Absorbance was examined at 515 nm wavelengths. Growth inhibition of 50% (IC_50_) was determined at the compound concentration resulting in 50% reduction of total protein increase in control cells. In clonogenic assay, cells were seeded in 6-well plates. After an eight-day treatment with the compound, cell colonies were rinsed with PBS, stained with 0.4% (*w/v*) crystal violet/20% methanol and lysed by 50 mM sodium citrate/50% ethanol. The plate was read by an enzyme-linked immunosorbent assay (ELISA) reader (595 nm) to get absorbance density values.

### 4.5. Cell Proliferation Assay with CFSE Staining

The cells were stained with CFSE at a final concentration of 5 μM at 37 °C for 10 min and cultured in 12-well plates in RPMI medium with 10% FBS at a density of 60,000 cells per well for 48 h. Cells were then cultured with 0.1% DMSO or the compound at 37 °C for another 24 or 48 h. After treatment, the cells were collected and detected using flow cytometric analysis (FACSan FL1 channel, Becton Dickinson, Mountain View, CA, USA). Proliferation index was analyzed with ModFit LT^TM^ 3.3 (Verity Software House, Topsham, ME, USA).

### 4.6. Cell Cycle Progression Analysis with PI Staining

After the treatment of the cells with the indicated compound for 24 or 48 h, the cells were harvested by trypsinization, fixed with 70% (*v/v*) alcohol at −20 °C for 30 min and washed with PBS. The cells were centrifuged and re-suspended with 0.5 mL PI solution containing Triton X-100 (0.1% *v/v*), RNase (100 μg/mL), and PI (80 μg/mL). DNA content was detected using flow cytometric analysis (FACSan FL2 channel and analyzed with BD CellQuest^TM^ Pro software (Becton Dickinson

### 4.7. Nucleosomal DNA Fragmentation Assay

Nucleosomal DNA fragmentation was determined using Cell Death Detection ELISA^PLUS^ kit (Roche, Mannheim, Germany) based on quantifying cytoplasmic histone-associated DNA fragments in cells after the induction of cell death. After the indicated treatment, the cells were lysed with lysis buffer and centrifuged. The supernatant was collected and incubated with HRP-conjugated anti-DNA-peroxidase antibody, following by washing and incubating with substrates of the antibody according to the manufacturer’s protocol. The plate was read by an ELISA reader (405 nm) to get the absorbance density values.

### 4.8. Western Bolting

After the treatment, cells were harvested, centrifuged and lysed with 50 μL ice-cold lysis buffer containing 20 mM Tris-HCl (pH 7.4), 150 mM NaCl, 1 mM EDTA, 1 mM EGTA, 1% Triton X-100, 1 mM β-glycerophosphate, 1 mM PMSF, 1 mM dithiothreitol, 10 μg/mL leupeptin, 1 mM NaF, and 1 mM Na_3_VO_4_ on ice for 20 min. Total protein was quantified, mixed with sample buffer and boiled at 95 °C for 8 min. Equal amount of protein (30 μg) was separated by electrophoresis in SDS-PAGE, transferred to PVDF membranes and detected with specific antibodies. The immunoreactive proteins after incubation with appropriately labeled secondary antibody were detected with an enhanced chemiluminescence detection kit (Amersham, Buckinghamshire, UK).

### 4.9. Immunofluorescence Confocal Microscopy Analysis

The cells were grown on coverslips in RPMI medium with 10% FBS. After the indicated treatment, the cells were washed with PBS, fixed with 100% methanol for 10 min, and then incubated with 0.1% Triton X-100 for 30 min. The reaction was blocked with 1% BSA in PBS. The cells were stained with anti-β-tubulin antibody (1:200 dilutions) for 40 min, FITC-conjugated secondary antibody (1:200 dilutions) for 1 h and 0.15 μg/mL DAPI for 5 min. The coverslips were air-dried and mounted on slides with ProLong^®^ Diamond Antifade Mountant (Thermo Fisher Scientific, MA, USA). The immunofluorescence images were captured by confocal microscope Zeiss LSM 880 (Carl Zeiss, Jena, Germany).

### 4.10. In Vitro Tubulin Polymerization Assay

Tubulin Polymerization Assay Kit (Cytoskeleton, CO, USA) was used in the study based on the demonstration that light is scattered by microtubules to a level which is proportional to the extent of microtubule polymer. After the incubation of tubulin proteins with the compound, the assessment was performed according to th manufacturer’s protocol. The absorbance was measured at a wavelength of 340 nm at 37 °C for 1 h continuously to detect the tubulin polymerization levels.

### 4.11. Microtubule Assembly Assay

After the treatment with the indicated agent for 6 h, the cells were harvested by trypsinization and collected by centrifugation. The cells were lysed with 200 μL of hypotonic buffer containing 20 mM Tris-HCl (pH 6.8), 1 mM MgCl_2_, 2 mM EGTA, 0.5% NP-40, 1 mM PMSF, and 1 mM dithiothreitol at 37 °C for 5 min. After centrifugation (15,000× *g*) at 25 °C for 15 min, the supernatant containing soluble form tubulins was obtained; the pellet containing particulate form polymerized tubulin was resuspended with 150 μL of hypotonic buffer and sonicated for 5 min. Tubulin contents in both fractions were detected by Western blotting.

### 4.12. Data Analysis

Data are presented as the mean  ± SEM for the indicated number of separate experiments. Student’s *t*-test is applied for comparison of two groups. *p*-values less than 0.05 are considered statistically significant.

## Figures and Tables

**Table 1:** The combination index (CI) of combination treatment between vinorelbine and YL-9 in NCI-H460 cells.

YL-9 (mM)	Vinorelbine (nM)	F	CI
10	1.3	0.08	0.66
10	2.5	0.34	0.46
10	5	0.69	0.38
10	10	0.86	0.43
10	20	0.92	0.55
30	1.3	0.06	0.79
30	2.5	0.5	0.31
30	5	0.78	0.29
30	10	0.92	0.29
30	20	0.96	0.39
50	1.3	0.12	0.49
50	2.5	0.54	0.28
50	5	0.8	0.27
50	10	0.94	0.25
50	20	0.94	0.47

Combination index (CI) values are calculated using Chou-Talalay method. Drug synergism, additive effect and antagonism are defined as CI value less than 1.0, equal to 1.0, or greater than 1.0, respectively.

## Supplementary Materials

The following are available online at https://www.mdpi.com/1422-0067/21/16/5608/s1, Figure S1: Effect of YL-9 on vinorelbine-mediated inhibition of colony formation, Figure S2: Effect of YL-9 on vinorelbine-induced activation of caspases, Figure S3: Effect of combinatory treatment on the exposure of N terminus of Bak, Figure S4: Examination of YL-9 stability in culture medium, Figure S5: Effect of YL-9 on vinorelbine-induced apoptosis in A549 cells.

## Abbreviations

CFSE	Carboxyfluorescein succinimidyl ester
CI	Combination index
FBS	Fetal bovine serum
NSCLC	Non-small cell lung cancer
PDE5	Phosphodiesterase type 5
PI	Propidium iodide
PMSF	Phenylmethylsulfonyl fluorid
ROS	Reactive oxygen species
SAC	Spindle assembly checkpoint
SRB	Sulforhodamine B

## References

[1] Piccirillo M.C., Daniele G., Di Maio M., Bryce J., De Feo G., Del Giudice A., Perrone F., Morabito A. (2010). Vinorelbine for non-small cell lung cancer. Expert Opin. Drug Saf..

[2] Kang D.H., Kim J.O., Jung S.S., Park H.S., Chung C., Park D., Lee J.E. (2019). Efficacy of Vinorelbine Monotherapy as Third-or Further-Line Therapy in Patients with Advanced Non-Small-Cell Lung Cancer. Oncology.

[3] Pilkington G., Boland A., Brown T., Oyee J., Bagust A., Dickson R. (2015). A systematic review of the clinical effectiveness of first-line chemotherapy for adult patients with locally advanced or metastatic non-small cell lung cancer. Thorax.

[4] Caffo O., Dipasquale M., Murgia V., Veccia A., Galligioni E. (2013). An evaluation of the pharmacokinetics and clinical use of vinorelbine for NSCLC treatment. Expert Opin. Drug Metab. Toxicol..

[5] Domvri K., Zarogoulidis K., Zogas N., Zarogoulidis P., Petanidis S., Porpodis K., Kioseoglou E., Hohenforst-Schmidt W. (2017). Potential synergistic effect of phosphodiesterase inhibitors with chemotherapy in lung cancer. J. Cancer.

[6] Kim J.Y., Son J.Y., Lee B.M., Kim H.S., Yoon S. (2018). Aging-Related Repositioned Drugs, Donepezil and Sildenafil Citrate, Increase Apoptosis of Anti-mitotic Drug-resistant KBV20C Cells Through Different Molecular Mechanisms. Anticancer Res..

[7] Chang J.F., Hsu J.L., Sheng Y.H., Leu W.J., Yu C.C., Chan S.H., Chan M.L., Hsu L.C., Liu S.P., Guh J.H. (2019). Phosphodiesterase Type 5 (PDE5) Inhibitors Sensitize Topoisomerase II Inhibitors in Killing Prostate Cancer Through PDE5-Independent Impairment of HR and NHEJ DNA Repair Systems. Front. Oncol..

[8] Chen L., Liu Y., Becher A., Diepold K., Schmid E., Fehn A., Brunner C., Rouhi A., Chiosis G., Cronauer M. (2020). Sildenafil triggers tumor lethality through altered expression of HSP90 and degradation of PKD2. Carcinogenesis.

[9] Dar M.I., Jan S., Reddy G.L., Wani R., Syed M., Dar M.J., Sawant S.D., Vishwakarma R.A., Syed S.H. (2020). Differentiation of human neuroblastoma cell line IMR-32 by sildenafil and its newly discovered analogue IS00384. Cell Signal..

[10] Li Q., Shu Y. (2014). Pharmacological modulation of cytotoxicity and cellular uptake of anti-cancer drugs by PDE5 inhibitors in lung cancer cells. Pharm. Res..

[11] Catalano S., Panza S., Augimeri G., Giordano C., Malivindi R., Gelsomino L., Marsico S., Giordano F., Győrffy B., Bonofiglio D. (2019). Phosphodiesterase 5 (PDE5) Is Highly Expressed in Cancer-Associated Fibroblasts and Enhances Breast Tumor Progression. Cancers.

[12] Li W.Q., Qureshi A.A., Robinson K.C., Han J. (2014). Sildenafil use and increased risk of incident melanoma in US men: A prospective cohort study. JAMA Intern. Med..

[13] Deng T., Duan X., Liu B., Lan Y., Cai C., Zhang T., Zhu W., Mai Z., Wu W., Zeng G. (2018). Association between phosphodiesterase type 5 inhibitors use and risk of melanoma: A meta-analysis. Neoplasma.

[14] Feng S., Zhou L., Liu Q., He Q., Liao B., Wei X., Li H., Wang K., Zhu Y. (2018). Are phosphodiesterase type 5 inhibitors associated with increased risk of melanoma? A systematic review and meta-analysis. Medicine.

[15] Lian Y., Yin H., Pollak M.N., Carrier S., Platt R.W., Suissa S., Azoulay L. (2016). Phosphodiesterase Type 5 Inhibitors and the Risk of Melanoma Skin Cancer. Eur. Urol..

[16] Pottegård A., Schmidt S.A., Olesen A.B., Achacoso N., Van Den Eeden S.K., Hallas J., Sørensen H.T., Friis S., Habel L.A. (2016). Use of sildenafil or other phosphodiesterase inhibitors and risk of melanoma. Br. J. Cancer.

[17] Dhayade S., Kaesler S., Sinnberg T., Dobrowinski H., Peters S., Naumann U., Liu H., Hunger R.E., Thunemann M., Biedermann T. (2016). Sildenafil Potentiates a cGMP-Dependent Pathway to Promote Melanoma Growth. Cell Rep..

[18] Arozarena I., Sanchez-Laorden B., Packer L., Hidalgo-Carcedo C., Hayward R., Viros A., Sahai E., Marais R. (2011). Oncogenic BRAF induces melanoma cell invasion by downregulating the cGMP-specific phosphodiesterase PDE5A. Cancer Cell.

[19] Kloner R.A. (2005). Pharmacology and drug interaction effects of the phosphodiesterase 5 inhibitors: Focus on alpha-blocker interactions. Am. J. Cardiol..

[20] Bolanos-Garcia V.M., Blundell T.L. (2011). BUB1 and BUBR1: Multifaceted kinases of the cell cycle. Trends Biochem. Sci..

[21] Shi Y., Fu X., Hua Y., Han Y., Lu Y., Wang J. (2012). The side population in human lung cancer cell line NCI-H460 is enriched in stem-like cancer cells. PLoS ONE.

[22] Zhu B., Lindsey A., Li N., Lee K., Ramirez-Alcantara V., Canzoneri J.C., Fajardo A., Madeira da Silva L., Thomas M., Piazza J.T. (2017). Phosphodiesterase 10A is overexpressed in lung tumor cells and inhibitors selectively suppress growth by blocking β-catenin and MAPK signaling. Oncotarget.

[23] Chou T.C. (2010). Drug combination studies and their synergy quantification using the Chou-Talalay method. Cancer Res..

[24] Toso R.J., Jordan M.A., Farrell K.W., Matsumoto B., Wilson L. (1993). Kinetic stabilization of microtubule dynamic instability in vitro by vinblastine. Biochemistry.

[25] Guo Y., Kim C., Ahmad S., Zhang J., Mao Y. (2012). CENP-E—Dependent BubR1 autophosphorylation enhances chromosome alignment and the mitotic checkpoint. J. Cell Biol..

[26] Wei L., Liang X.W., Zhang Q.H., Li M., Yuan J., Li S., Sun S.C., Ouyang Y.C., Schatten H., Sun Q.Y. (2010). BubR1 is a spindle assembly checkpoint protein regulating meiotic cell cycle progression of mouse oocyte. Cell Cycle.

[27] Eichhorn J.M., Sakurikarm N., Alford S.E., Chu R., Chambers T.C. (2013). Critical role of anti-apoptotic Bcl-2 protein phosphorylation in mitotic death. Cell Death Dis..

[28] Griffiths G.J., Corfe B.M., Savory P., Leech S., Esposti M.D., Hickman J.A., Dive C. (2001). Cellular damage signals promote sequential changes at the N-terminus and BH-1 domain of the pro-apoptotic protein Bak. Oncogene.

[29] Gadde S., Heald R. (2004). Mechanisms and molecules of the mitotic spindle. Curr. Biol..

[30] Westermann S., Weber K. (2003). Post-translational modifications regulate microtubule function. Nat. Rev. Mol. Cell Biol..

[31] Mohan R., Panda D. (2008). Kinetic stabilization of microtubule dynamics by estramustine is associated with tubulin acetylation, spindle abnormalities, and mitotic arrest. Cancer Res..

[32] Skibbens R.V., Skeen V.P., Salmon E.D. (1993). Directional instability of kinetochore motility during chromosome congression and segregation in mitotic newt lung cells: A push-pull mechanism. J. Cell Biol..

[33] Ngan V.K., Bellman K., Panda D., Hill B.T., Jordan M.A., Wilson L. (2000). Novel actions of the antitumor drugs vinflunine and vinorelbine on microtubules. Cancer Res..

[34] Eshun-Wilson L., Zhang R., Portran D., Nachury M.V., Toso D.B., Löhr T., Vendruscolo M., Bonomi M., Fraser J.S., Nogales E. (2019). Effects of α-tubulin acetylation on microtubule structure and stability. Proc. Natl. Acad. Sci. USA.

[35] Hinchcliffe E.H., Sluder G. (2001). “It takes two to tango”: Understanding how centrosome duplication is regulated throughout the cell cycle. Genes Dev..

[36] Saunders W.S., Shuster M., Huang X., Gharaibeh B., Enyenihi A.H., Petersen I., Gollin S.M. (2000). Chromosomal instability and cytoskeletal defects in oral cancer cells. Proc. Natl. Acad. Sci. USA.

[37] Quintyne N.J., Reing J.E., Hoffelder D.R., Gollin S.M., Saunders W.S. (2005). Spindle multipolarity is prevented by centrosomal clustering. Science.

[38] Saunders W. (2005). Centrosomal amplification and spindle multipolarity in cancer cells. Semin. Cancer Biol..

[39] Zeng X., Xu W.K., Lok T.M., Ma H.T., Poon R.Y.C. (2019). Imbalance of the spindle-assembly checkpoint promotes spindle poison-mediated cytotoxicity with distinct kinetics. Cell Death Dis..

[40] Brito D.A., Yang Z., Rieder C.L. (2008). Microtubules do not promote mitotic slippage when the spindle assembly checkpoint cannot be satisfied. J. Cell Biol..

[41] Park Y.Y., Ahn J.H., Cho M.G., Lee J.H. (2018). ATP depletion during mitotic arrest induces mitotic slippage and APC/C_Cdh1_-dependent cyclin B1 degradation. Exp. Mol. Med..

[42] Balachandran R.S., Kipreos E.T. (2017). Addressing a weakness of anticancer therapy with mitosis inhibitors: Mitotic slippage. Mol. Cell Oncol..

[43] Jablonski S.A., Chan G.K., Cooke C.A., Earnshaw W.C., Yen T.J. (1998). The hBUB1 and hBUBR1 kinases sequentially assemble onto kinetochores during prophase with hBUBR1 concentrating at the kinetochore plates in mitosis. Chromosoma.

[44] Li W., Lan Z., Wu H., Wu S., Meadows J., Chen J., Zhu V., Dai W. (1999). BUBR1 phosphorylation is regulated during mitotic checkpoint activation. Cell Growth Differ..

[45] Ditchfield C., Johnson V.L., Tighe A., Ellston R., Haworth C., Johnson T., Mortlock A., Keen N., Taylor S.S. (2003). Aurora B couples chromosome alignment with anaphase by targeting BubR1, Mad2, and Cenp-E to kinetochores. J. Cell Biol..

[46] Matsumura S., Toyoshima F., Nishida E. (2007). Polo-like kinase 1 facilitates chromosome alignment during prometaphase through BubR1. J. Biol. Chem..

[47] Huang H., Hittle J., Zappacosta F., Annan R.S., Hershko A., Yen T.J. (2008). Phosphorylation sites in BubR1 that regulate kinetochore attachment, tension, and mitotic exit. J. Cell Biol..

[48] Kim M., Murphy K., Liu F., Parker S.E., Dowling M.L., Baff W., Kao G.D. (2005). Caspase-Mediated specific cleavage of BubR1 is a determinant of mitotic progression. Mol. Cell Biol..

