# Supplementary material for: Discovery of Novel Agents on Spindle Assembly Checkpoint to Sensitize Vinorelbine-Induced Mitotic Cell Death against Human Non-Small Cell Lung Cancers
Source: Int J Mol Sci. 2020 Aug 5;21(16):5608. doi: 10.3390/ijms21165608 (PMC7460560; doi:10.3390/ijms21165608)
Supplement: Supplementary file 1 [file ijms-21-05608-s001.zip › YL-9 HPLC.pdf]

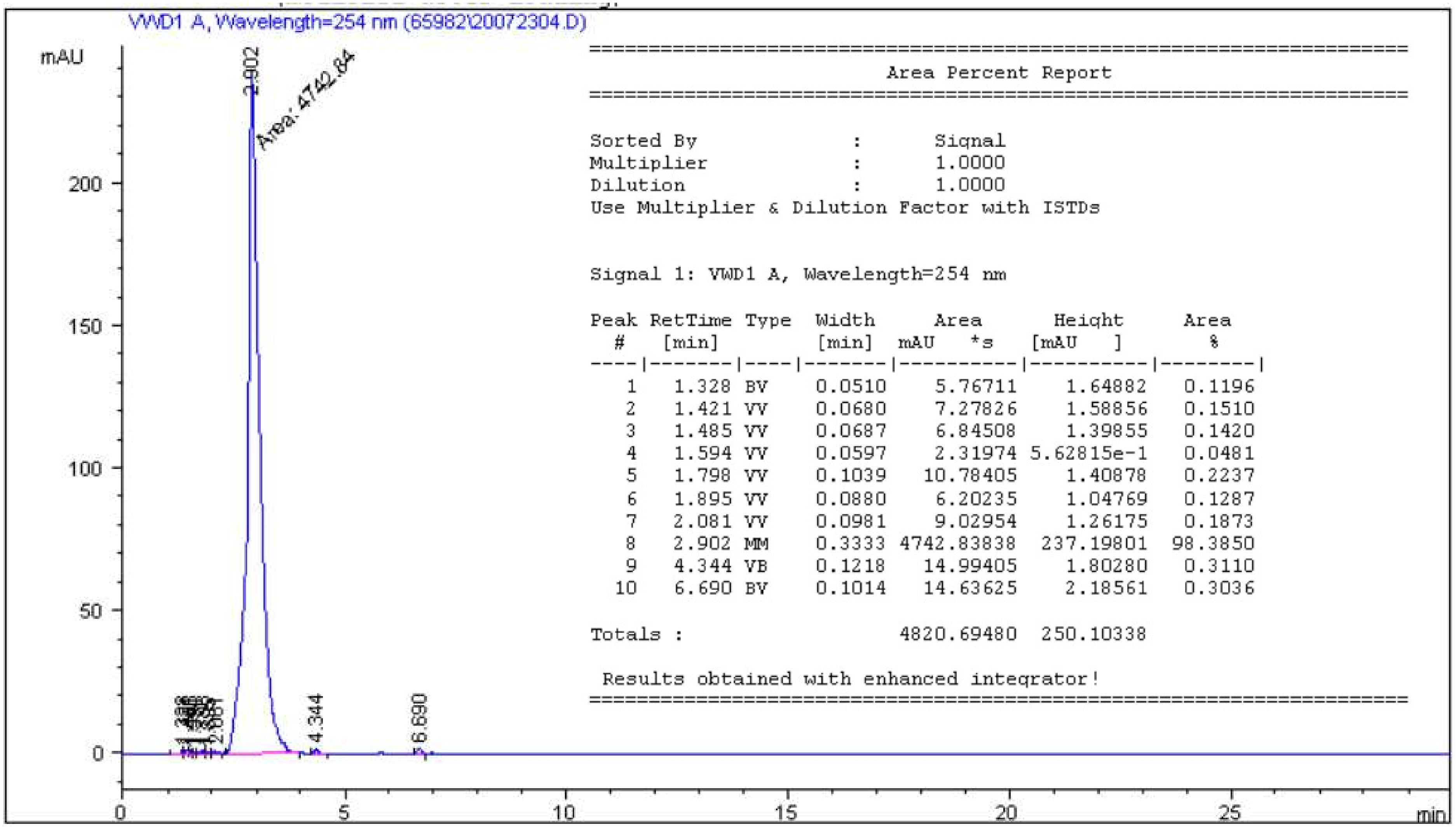

Area Percent Report

Sorted By : Signal
Multiplier : 1.0000
Dilution : 1.0000
Use Multiplier & Dilution Factor with ISTDs

Signal 1: VWD1 A, Wavelength=254 nm

Peak #	RetTime [min]	Type	Width [min]	Area mAU *s	Height [mAU]	Area %
1	1.328	BV	0.0510	5.76711	1.64882	0.1196
2	1.421	VV	0.0680	7.27826	1.58856	0.1510
3	1.485	VV	0.0687	6.84508	1.39855	0.1420
4	1.594	VV	0.0597	2.31974	5.62815e-1	0.0481
5	1.798	VV	0.1039	10.78405	1.40878	0.2237
6	1.895	VV	0.0880	6.20235	1.04769	0.1287
7	2.081	VV	0.0981	9.02954	1.26175	0.1873
8	2.902	MM	0.3333	4742.83838	237.19801	98.3850
9	4.344	VB	0.1218	14.99405	1.80280	0.3110
10	6.690	BV	0.1014	14.63625	2.18561	0.3036
Totals :				4820.69480	250.10338	

Results obtained with enhanced integrator!

HPLC analysis of YL-9

Column: Agilent ZORBAX Eclipse XDB-C8, 80Å, 4.6 x 150 mm, 5 µm.

Eluent: 60% acetonitrile and 40% water

Flow rate: 1 mL/min

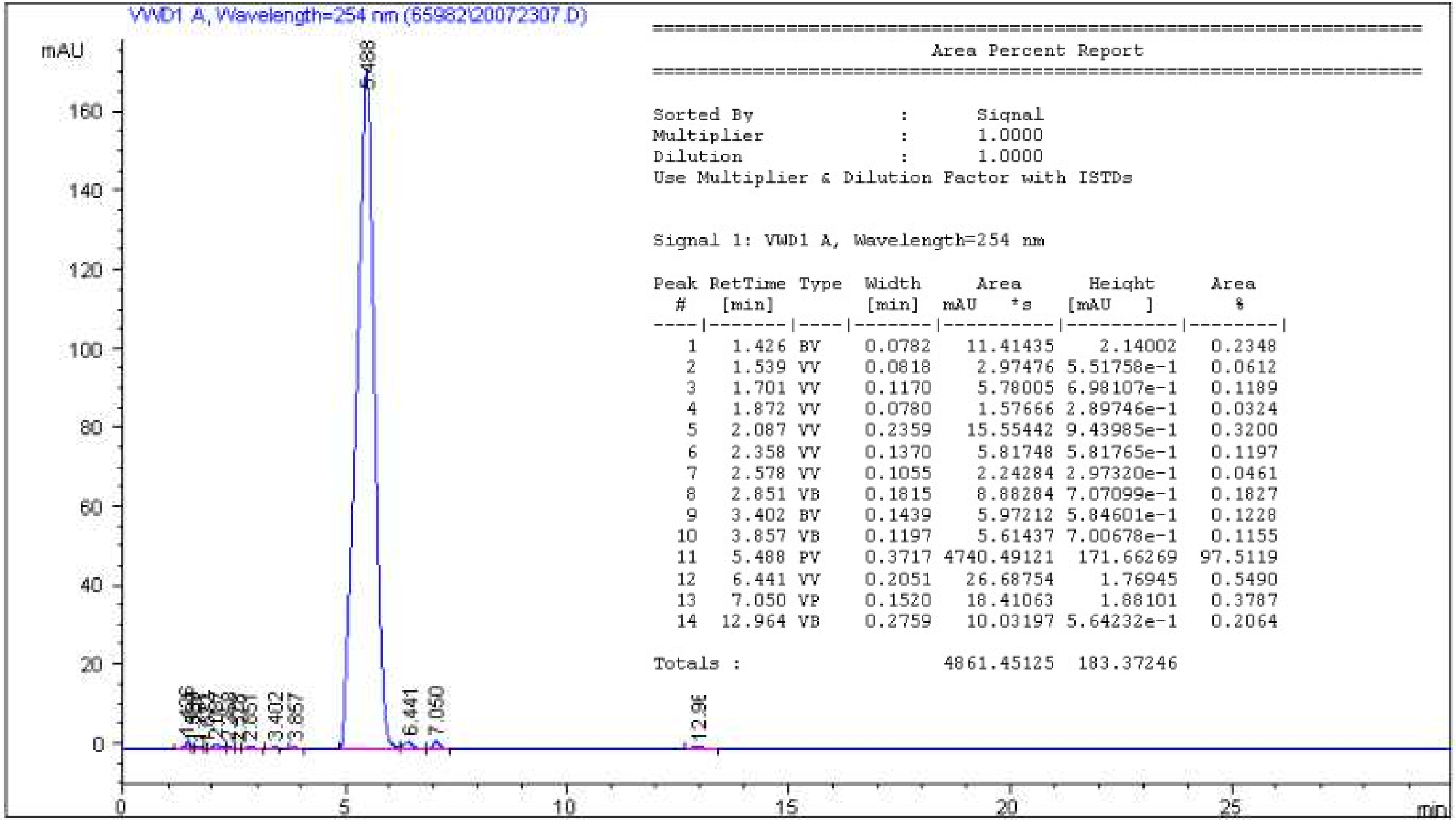

Area Percent Report

Sorted By : Signal
Multiplier : 1.0000
Dilution : 1.0000
Use Multiplier & Dilution Factor with ISTDs

Signal 1: VWD1 A, Wavelength=254 nm

Peak #	RetTime [min]	Type	Width [min]	Area mAU *s	Height [mAU]	Area %
1	1.426	BV	0.0782	11.41435	2.14002	0.2348
2	1.539	VV	0.0818	2.97476	5.51758e-1	0.0612
3	1.701	VV	0.1170	5.78005	6.98107e-1	0.1189
4	1.872	VV	0.0780	1.57666	2.89746e-1	0.0324
5	2.087	VV	0.2359	15.55442	9.43985e-1	0.3200
6	2.358	VV	0.1370	5.81748	5.81765e-1	0.1197
7	2.578	VV	0.1055	2.24284	2.97320e-1	0.0461
8	2.851	VB	0.1815	8.88284	7.07099e-1	0.1827
9	3.402	BV	0.1439	5.97212	5.84601e-1	0.1228
10	3.857	VB	0.1197	5.61437	7.00678e-1	0.1155
11	5.488	PV	0.3717	4740.49121	171.66269	97.5119
12	6.441	VV	0.2051	26.68754	1.76945	0.5490
13	7.050	VP	0.1520	18.41063	1.88101	0.3787
14	12.964	VB	0.2759	10.03197	5.64232e-1	0.2064
Totals :				4861.45125	183.37246	

HPLC analysis of YL-9

Column: Agilent ZORBAX Eclipse XDB-C8, 80Å, 4.6 x 150 mm, 5 µm.

Eluent: 40% acetonitrile and 60% water

Flow rate: 1 mL/min
