# Supplementary material for: Discovery of Novel Agents on Spindle Assembly Checkpoint to Sensitize Vinorelbine-Induced Mitotic Cell Death against Human Non-Small Cell Lung Cancers
Source: Int J Mol Sci. 2020 Aug 5;21(16):5608. doi: 10.3390/ijms21165608 (PMC7460560; doi:10.3390/ijms21165608)
Supplement: Supplementary file 1 [file ijms-21-05608-s001.zip › YL-9 NMR.pdf]

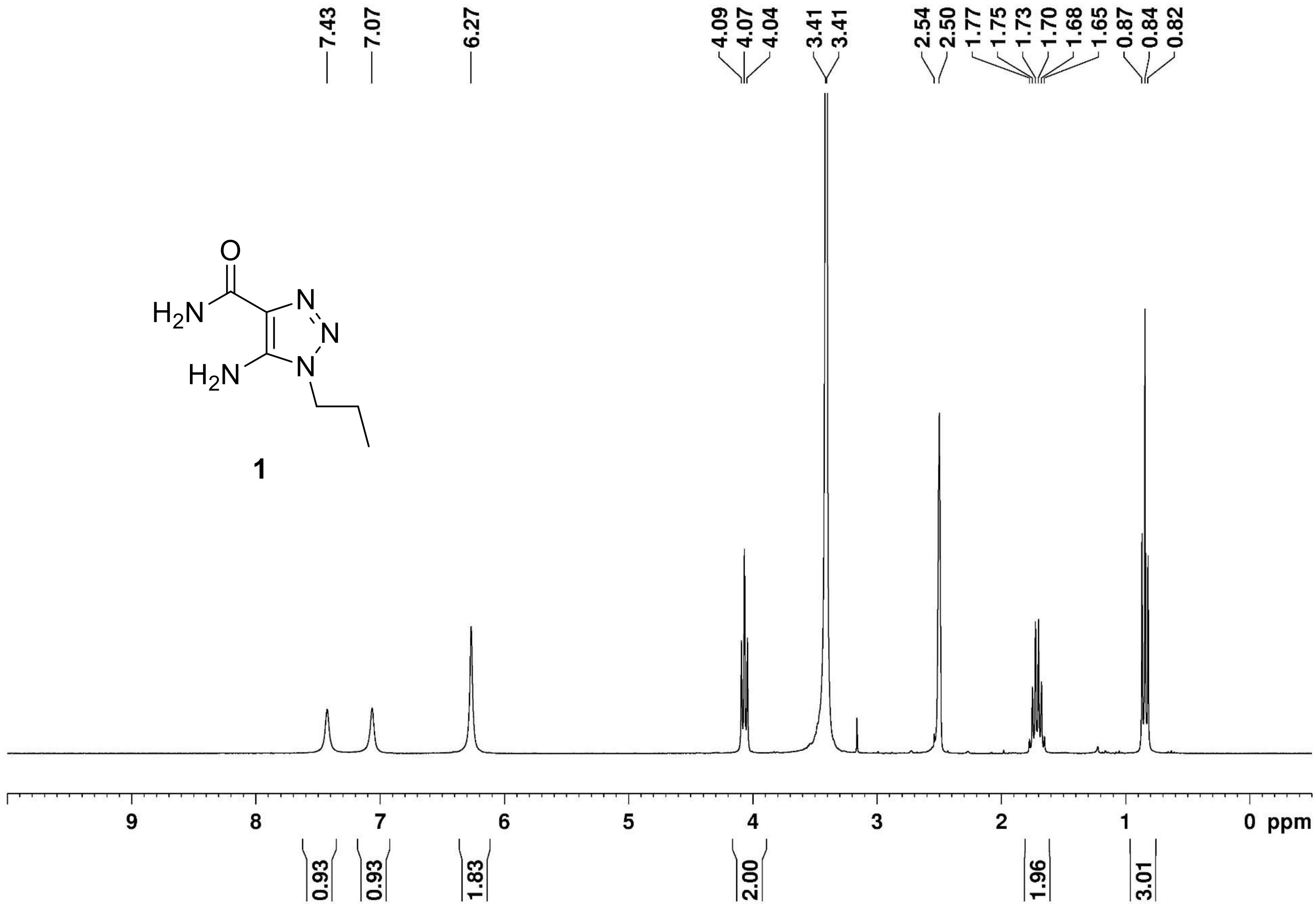


$^{1}H$ NMR of compound **1** (300 MHz DMSO-$d_6$)

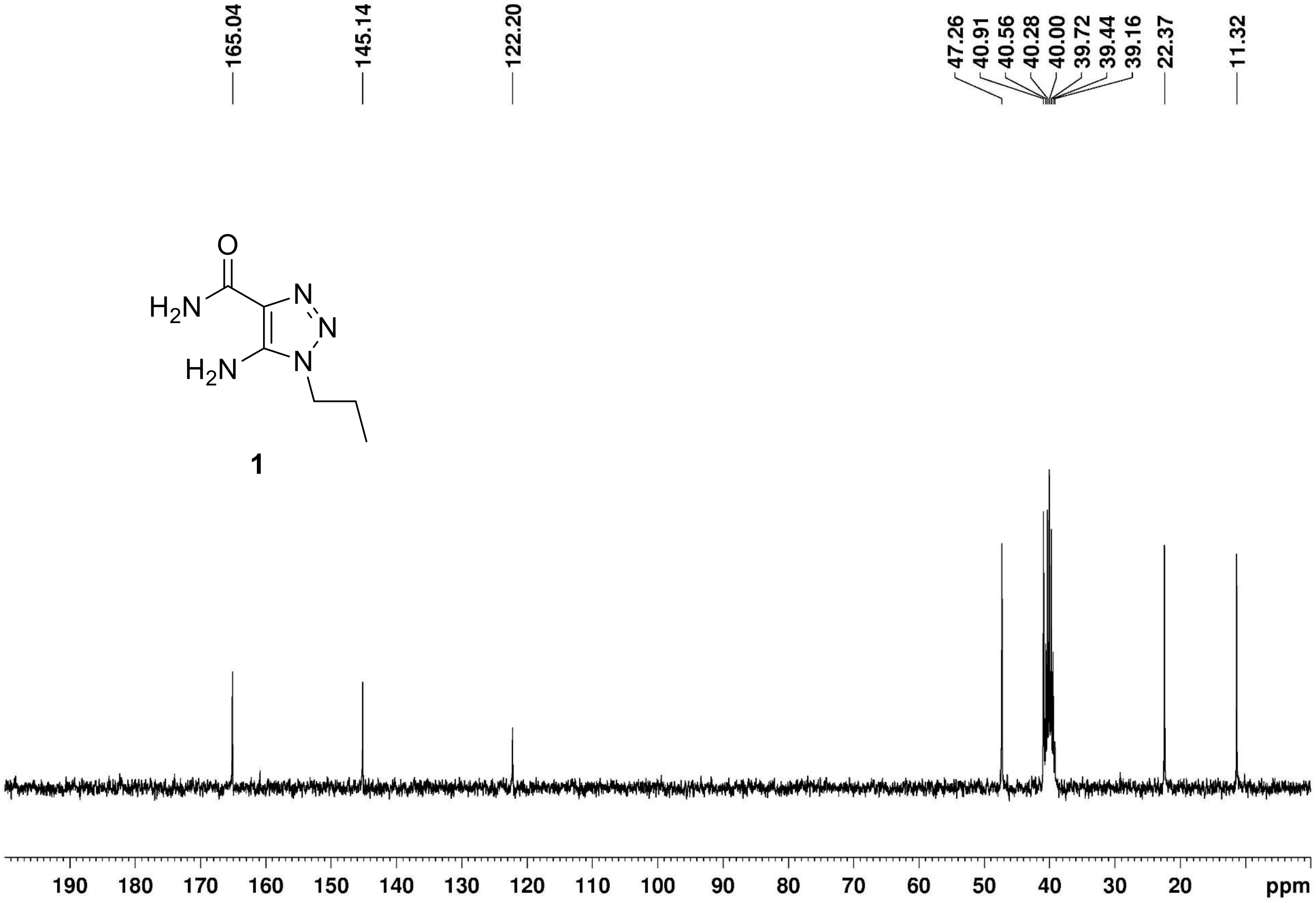


$^{13}C\{^1H\}$ NMR of compound **1** (75 MHz, DMSO-$d_6$)

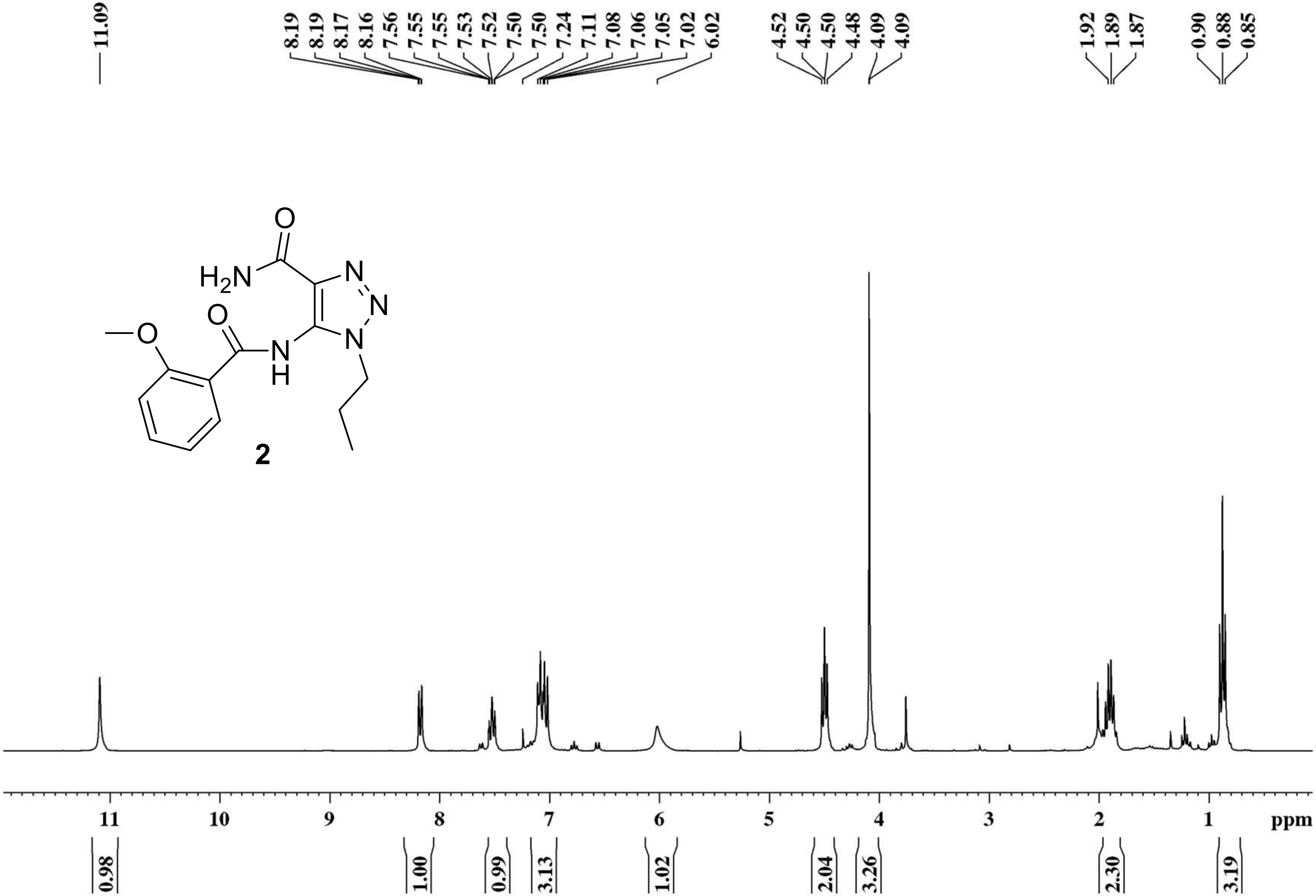


$^{1}$H NMR of compound **2** (300 MHz $CDCl_3$)

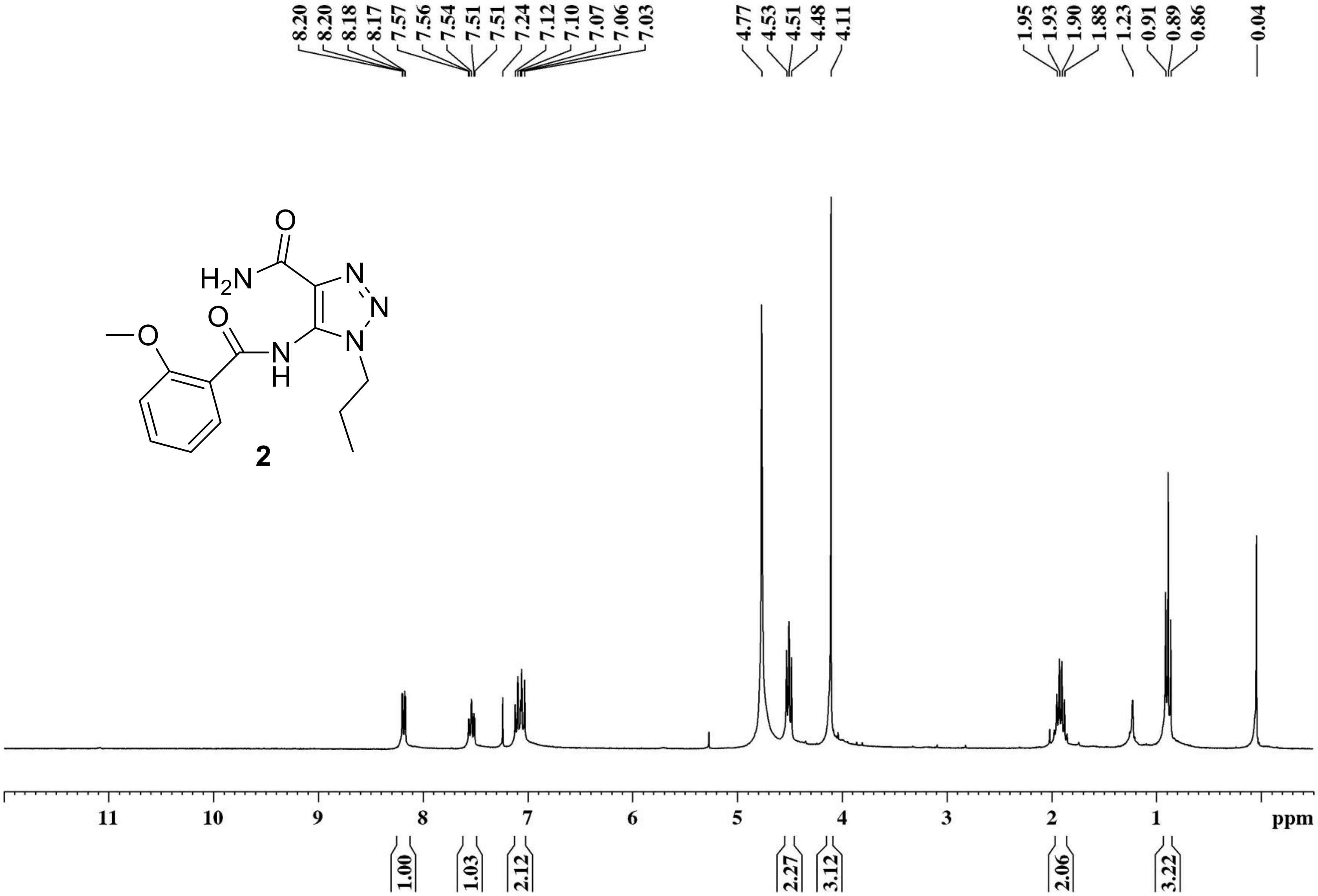


$^1$H NMR of compound **2 + D$_2$O** (300 MHz $CDCl_3$)

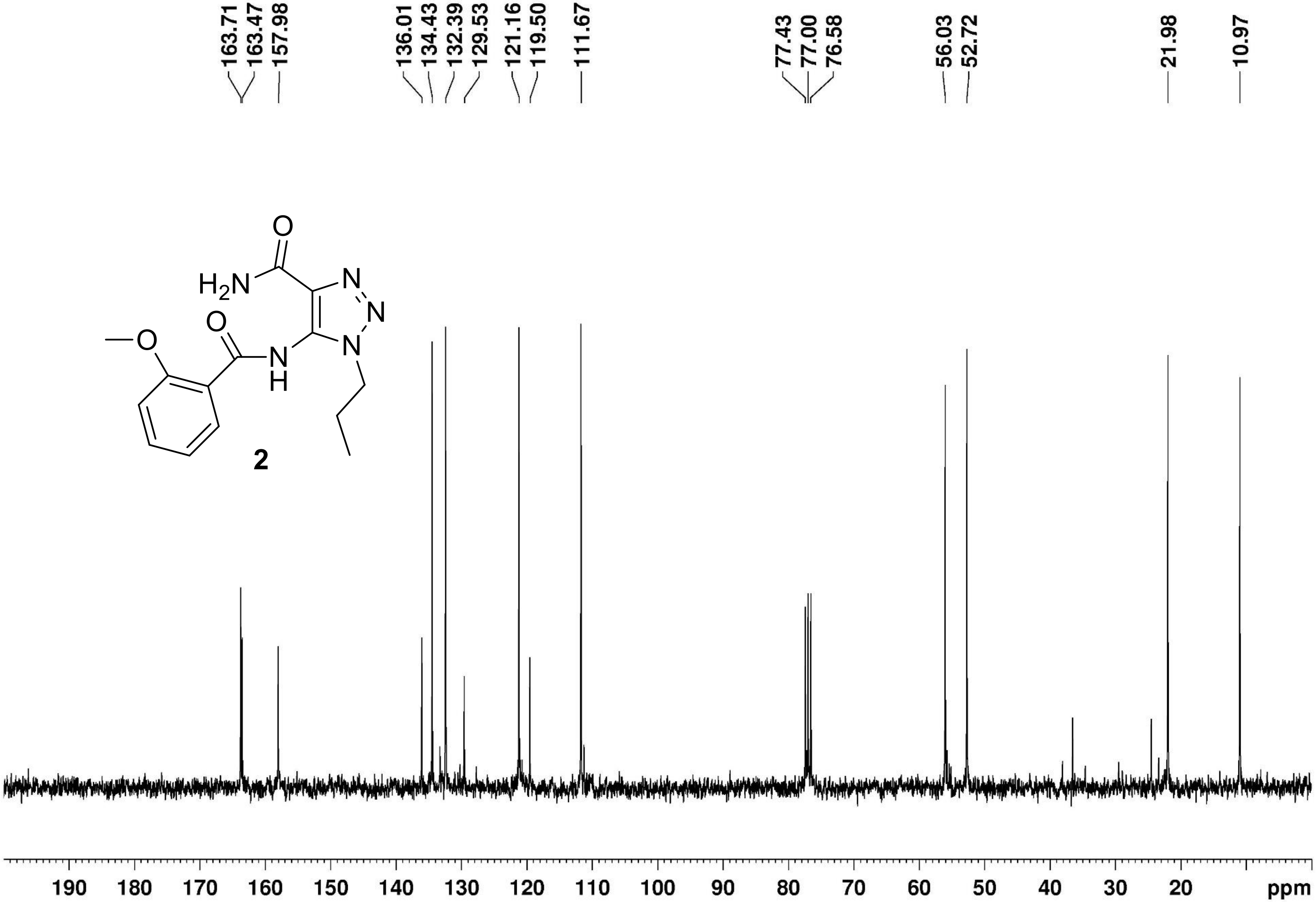


$^{13}C\{^1H\}$ NMR of compound **2** (75 MHz, $CDCl_3$)

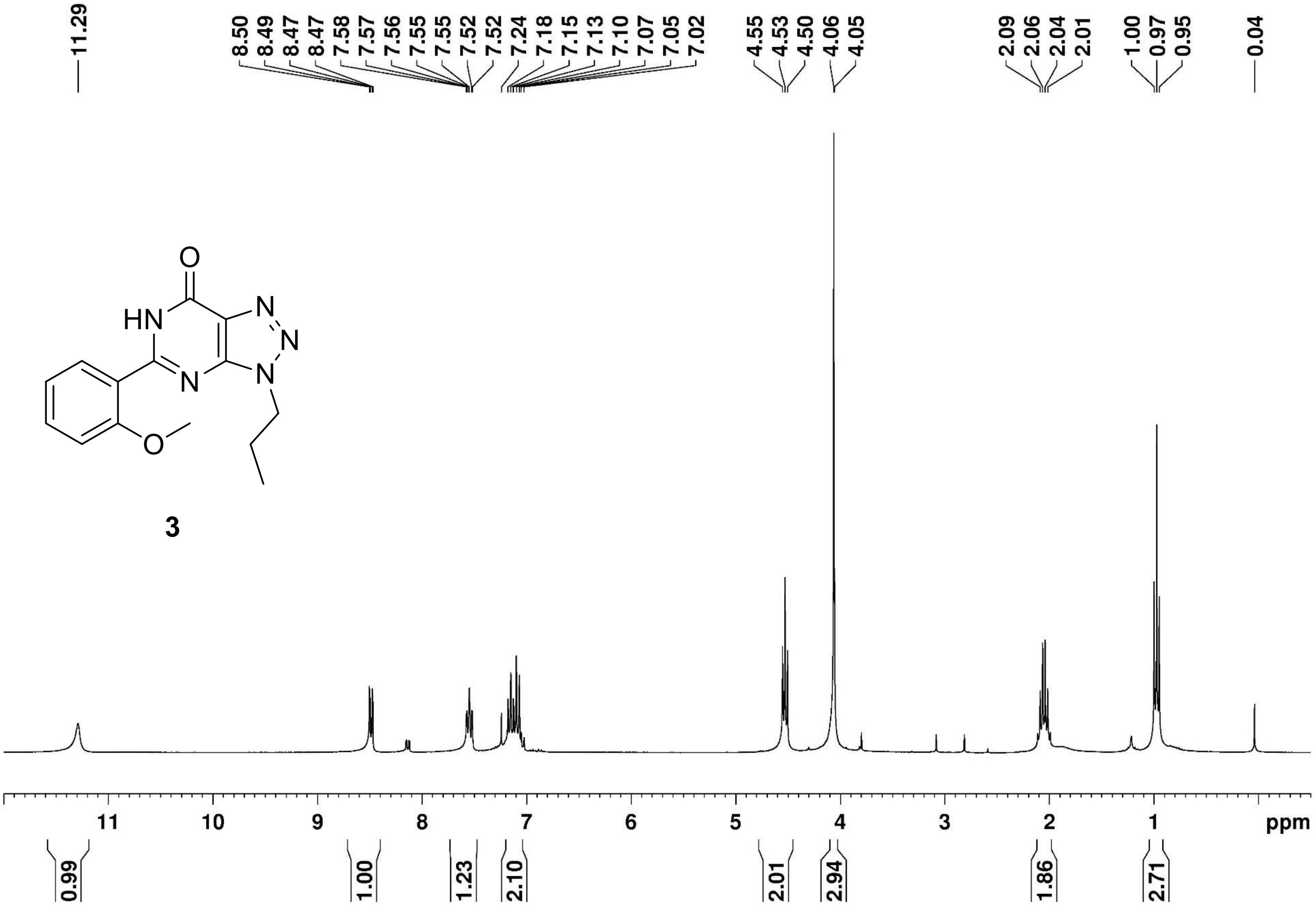


$^{1}$H NMR of compound **3** (300 MHz $CDCl_3$)

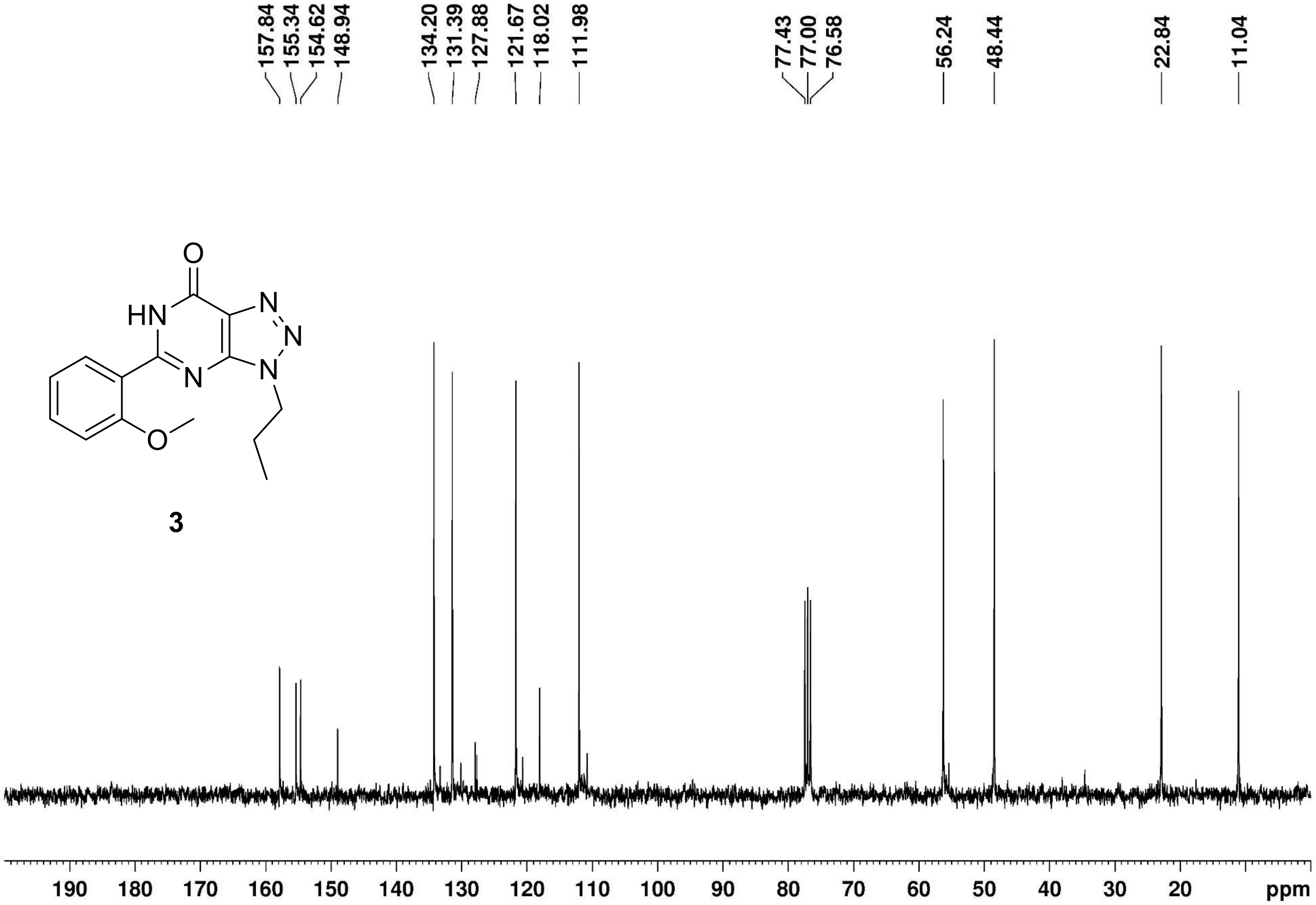


$^{13}C$ {$^{1}H$} NMR of compound **3** (75 MHz, $CDCl_3$)

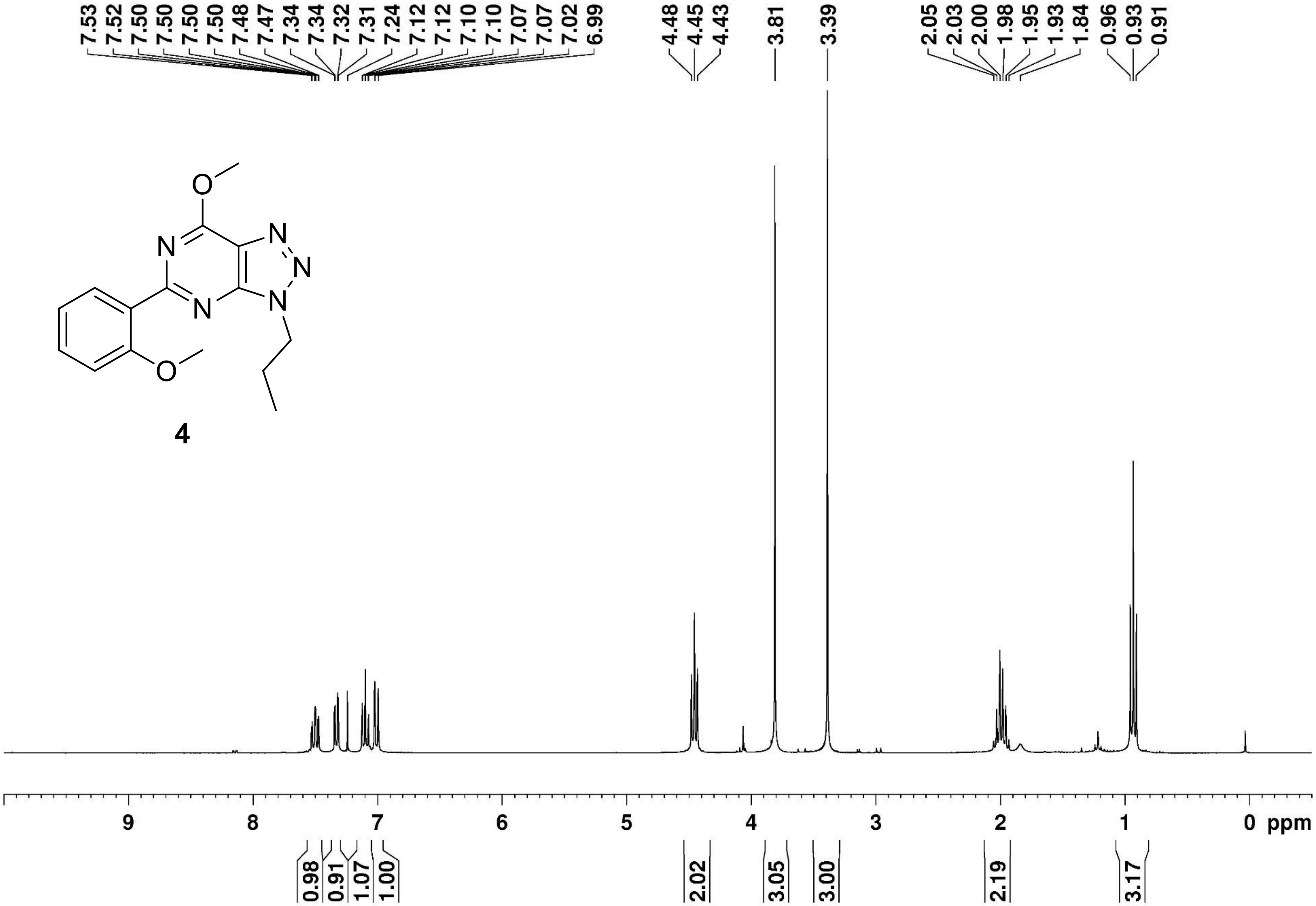


$^{1}$H NMR of compound **4** (300 MHz $CDCl_3$)

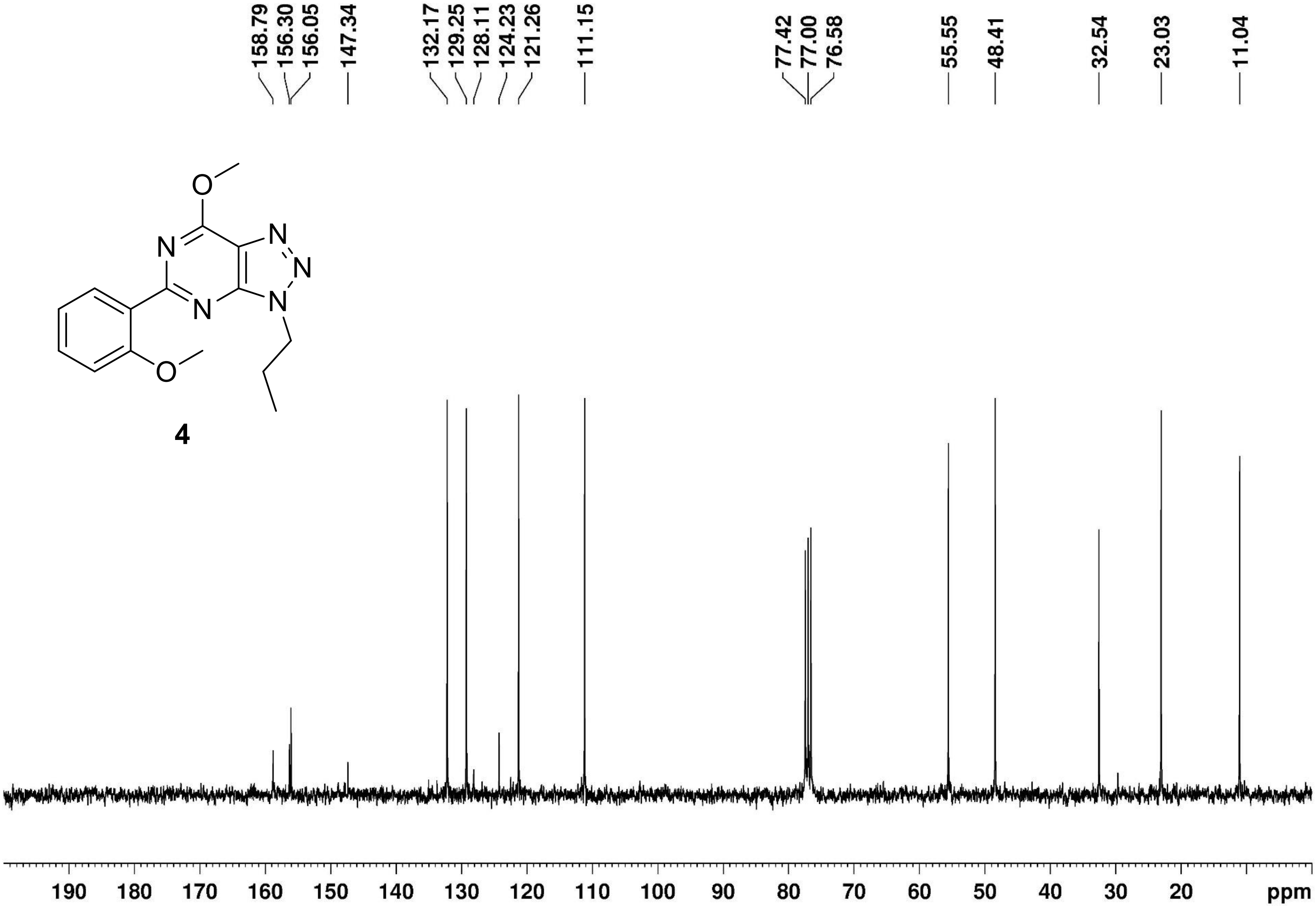


$^{13}C\{^{1}H\}$ NMR of compound **4** (75 MHz $CDCl_3$)

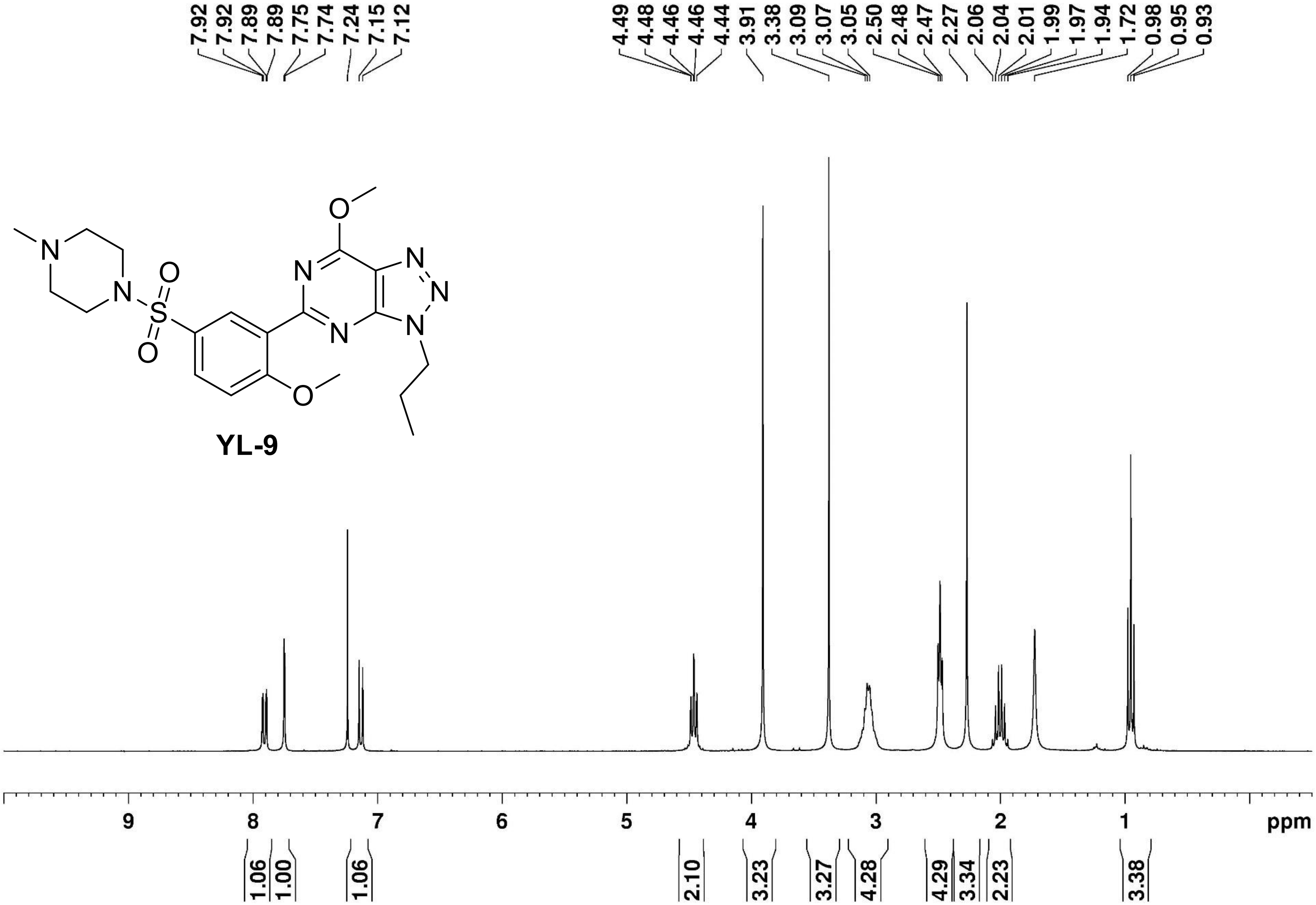


$^{1}$H NMR of compound **YL-9** (300 MHz $CDCl_3$)

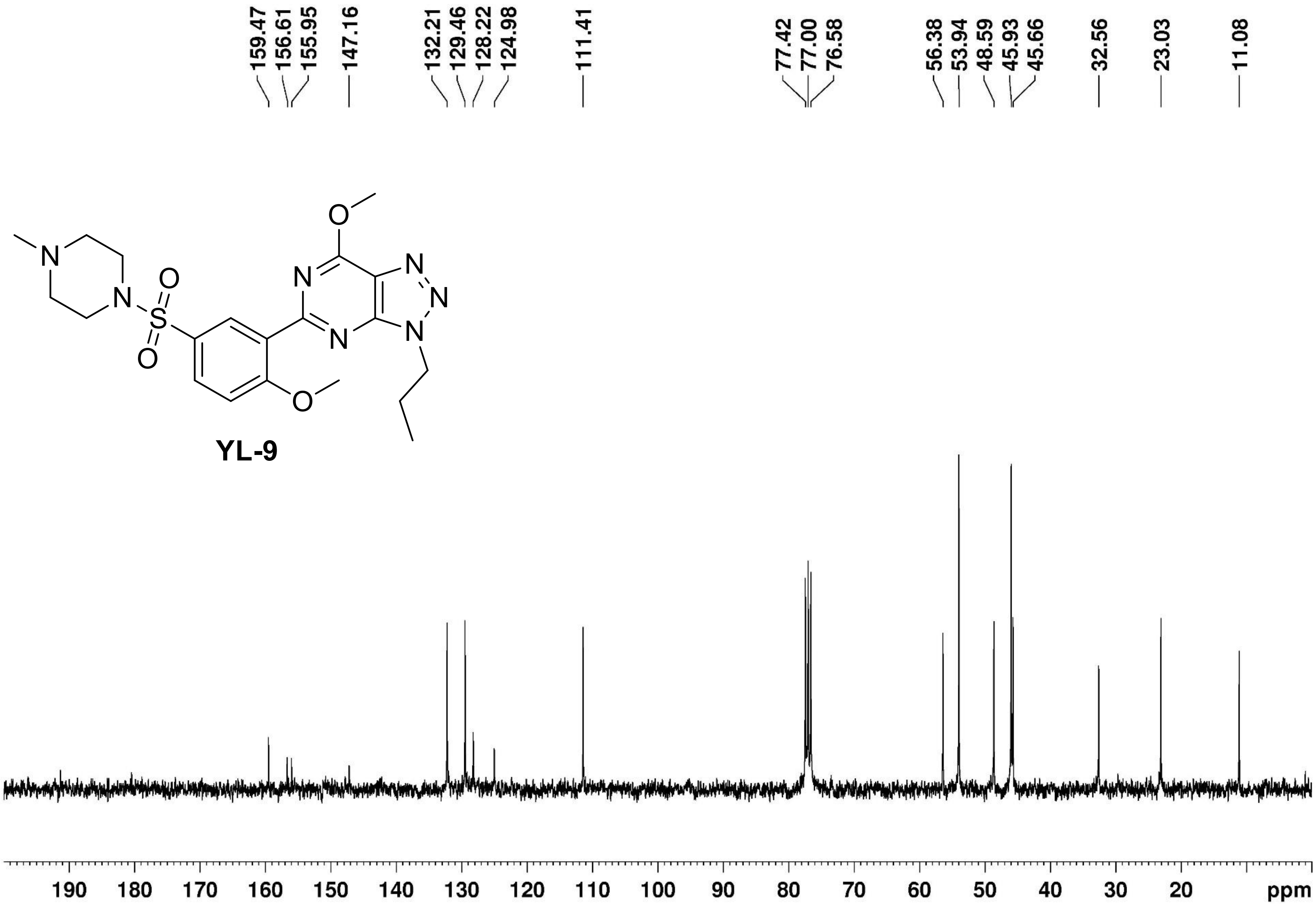


$^{13}C\{^{1}H\}$ NMR of compound **YL-9** (75 MHz $CDCl_3$)
