# Supplementary material for: Discovery of Novel Agents on Spindle Assembly Checkpoint to Sensitize Vinorelbine-Induced Mitotic Cell Death against Human Non-Small Cell Lung Cancers
Source: Int J Mol Sci. 2020 Aug 5;21(16):5608. doi: 10.3390/ijms21165608 (PMC7460560; doi:10.3390/ijms21165608)
Supplement: Supplementary file 1 [file ijms-21-05608-s001.zip › Supplementary material - Synthesis of YL-9.docx]

Synthesis of **YL-9**

**Scheme 1.** Chemical synthesis of **YL-9**

**General information.** Dichloromethane was distilled from calcium hydride; tetrahydrofuran and diethyl ether were distilled from sodium/benzophenone. Reagents were purchased from commercial sources and used without further purification. Chemical shifts for ^1^H NMR and ^13^C NMR spectra are reported in δ units (parts per million) with reference to residual solvent peaks. The multiplicities are abbreviated as follows: s = singlet, d = doublet, t = triplet, q = quartet, m = multiplet, dd = doublet of doublets, td = triplet of doublets. Thin-layer chromatography (TLC) was conducted using precoated silica gel 60 F254 plates containing a fluorescent indicator and TLC spots were examined under UV light or revealed by KMnO_4_ solution. Column purification was conducted using silica gel (230−400 mesh). Microwave-assisted reactions were performed in a CEM Discover single-mode microwave reactor using a sealed reaction vessel (10 mL, max pressure = 30 bar), equipped with a vertically focused IR temperature sensor; controlled temperature, power, and time settings were used for all reactions. High-resolution mass spectrometry (HRMS) data were recorded on a JMS-700 quadrupole mass spectrometer.

**5-Amino-1-propyl-1*H*-1,2,3-triazole-4-carboxamide** (**1**)^1^

Sodium azide (6.30 g, 0.097 mol) was added to a solution of 1-bromopropane (6.01 g, 0.049 mol) and dimethyl sulfoxide (DMSO, 40 mL). The reaction mixture was heated in an oil bath (65 °C) for 2 h, then added with 2-cyanoacetamide (2.01 g, 0.024 mol) and sodium ethoxide (4.90 g, 0.072 mol), heated at 65 °C for another 18 h, quenched with water (100 mL) and extracted with ethyl acetate (40 mL × 3). The combined organic layers were dried over sodium sulfate, filtered and concentrated. The crude product was recrystallized in methanol to give **1** (3.00 g, 0.018 mol, 74%) as a white solid. Mp 191.0-192.0 °C; ^1^H NMR (DMSO-*d*_6_, 300 MHz) δ 0.84 (*t*, *J* = 7.5Hz, 3H), 1.65‒1.77 (m, 2H), 4.07 (*t*, *J* = 7.5 Hz, 2H), 6.27 (s, 2H), 7.07 (s, 1H), 7.43 (s, 1H); ^13^C{^1^H} NMR (DMSO-*d*_6_, 75 MHz) δ 11.3, 22.4, 47.3, 122.2, 145.1, 165.0; HRMS (ESI) calcd for [M]^+^ (C_6_H_11_N_5_O) 169.0964; found 169.0958.

**5-(2-Methoxybenzamido)-1-propyl-1*H*-1,2,3- triazole-4- carboxamide** (**2**)

2-Methoxybenzoyl chloride (106.0 mg, 0.62 mmol) dissolved in dichloromethane (2 mL) was added to a solution of compound **1**(70.0 mg, 0.41 mmol), 4-(dimethylamino)pyridine (DMAP, 5.1 mg, 0.041mmol) and pyridine (1.6 mL) in a microwave reaction vessel (10 mL). The reaction mixture was heated in a microwave ~~oven~~ synthesizer (100 °C) for 30 min, diluted with water (10 mL) and extracted with diethyl ether (10 mL × 3). The combined organic layers were washed with saturated NaCl_(aq)_ (8 mL), dried over sodium sulfate, filtered and concentrated. The crude product was purified by column chromatography (SiO_2_, ethyl acetate/hexanes, 1:3; *R_f_* 0.29) to give **2** as a white solid (79.7 mg, 0.26 mmole, 64%). Mp 115.5‒116.0 °C; ^1^H NMR (CDCl_3_, 300 MHz) δ 0.88 (*t*, *J* = 7.4 Hz, 3H), 1.85‒1.97 (m, 2H), 4.10 (s, 3H), 4.50 (*t*, *J* = 7.3 Hz, 2H), 6.02 (s, 1H), 7.00‒7.12 (m, 3H), 7.53 (dt, *J* = 7.8 Hz, *J* = 1.7 Hz, 1H), 8.19 (dd, *J* = 7.8 Hz, *J* = 1.7 Hz, 1H), 11.09 (s, 1H); ^13^C{^1^H} NMR (CDCl_3_, 75 MHz) δ 11.0, 22.0, 52.7, 56.0, 111.7, 119.5, 121.2, 129.5, 132.4, 134.4, 136.0, 158.0, 163.5, 163.7; HRMS (ESI) calcd for [M - H]^-^ (C_14_H_16_N_5_O_3_) 302.1253; found 302.1248.

**5-(2-Methoxyphenyl)-3-propyl-3*H*-[1,2,3]triazolo[4,5-d]pyrimidin-7(6H)-one** (**3**)

A reaction mixture of compound **2** (100.0 mg, 0.33 mmol), KOH_(aq)_ (10% w/w, 3 mL) and ethanol (1.2 mL) was heated in a microwave ~~oven~~ synthesizer (130 °C) for 1 h, cooled to rt, acidified with HCl_(aq)_ (1*N*, 5 mL) and concentrated. The crude product was purified by column chromatography (SiO_2_, ethyl acetate/chloroform, 1:1; *R_f_* 0.50) to give **3** as a white solid (64.0 mg, 0.23 mmole, 68%). Mp 158.0‒159.0 ℃; ^1^H NMR (CDCl_3_, 300 MHz) δ 0.97 (t, *J* = 7.4 Hz, 3H), 2.02‒2.09 (m, 2H), 4.07 (s, 3H), 4.53 (t, *J* = 7.2 Hz, 2H), 7.05‒7.18 (m, 2H), 7.56 (dt, *J* = 7.9 Hz, *J* = 1.8 Hz, 1H), 8.51 (dd, *J* = 7.8 Hz, *J* = 1.7 Hz, 1H), 11.29 (s, 1H); ^13^C{^1^H} NMR (CDCl_3_, 75 MHz) δ 11.0, 22.8, 48.4, 56.2, 112.0, 118.0, 121.7, 127.9, 131.4, 134.2, 148.9, 154.6, 155.3, 157.8; HRMS (ESI) calcd for [M - H]^-^ (C_14_H_14_N_5_O_2_) 284.1147; found 284.1141.

**7-Methoxy-5-(2-methoxyphenyl)-3-propyl-3*H*-[1,2,3]triazolo[4,5-d]pyrimidine** (**4**)

A solution of compound **3** (40.0 mg, 0.14 mmol) in dimethylformamide (DMF, 1 mL) was added into a reaction flask containing sodium hydride (16.9 mg, 0.70 mmol) at 0 °C. The reaction mixture was stirred at 25 °C for 30 min, added with iodomethane (87 μL, 0.70 mmol), stirred at 25 °C for another 1 h, quenched with ethanol (1 mL) concentrate and diluted with ethyl acetate (15 mL). The organic solution was washed with water (5 mL × 2) and saturated NaCl_(aq)_ (3 mL), dried over sodium sulfate, filtered and concentrated. The crude product was purified by column chromatography (SiO_2_, ethyl acetate/hexanes, 1:1; *R_f_* 0.50) to give **4** as a white solid (21.0 mg, 0.072 mmol, 50%). Mp: 150.0‒151.5℃; ^1^H NMR (CDCl_3_, 300 MHz) δ 0.93 (t, *J* = 7.4 Hz, 3H), 1.84‒2.05 (m, 2H), 3.39 (s, 3H), 3.81 (s, 3H), 4.45 (t, *J* = 7.2 Hz, 2H), 7.01 (d, *J* = 8.4 Hz, 1H), 7.10 (dt, *J* = 7.4 Hz, *J* = 0.8 Hz, 1H), 7.33 (dt, *J* = 7.5 Hz, *J* = 1.7 Hz, 1H), 7.50 (dt, *J* = 7.9 Hz, *J* = 1.7 Hz, 1H); ^13^C{^1^H} NMR (CDCl_3_, 75 MHz) δ 11.0, 23.0, 32.5, 48.4, 55.6, 111.2, 121.3, 124.2, 128.1, 129.2, 132.2, 147.3, 156.1, 156.3, 158.8; HRMS (ESI) calcd for [M + Na]^+^ (C_15_H_17_N_5_O_2_Na) 322.1280, found 322.1271.

**7-Methoxy-5-(2-methoxy-5-((4-methylpiperazin-1-yl)sulfonyl)phenyl)-3-propyl-3*H*-[1,2,3]triazolo[4,5-d]pyrimidine** (**YL-9**)

Chlorosulfonic acid (0.11 mL, 1.59 mmol) was added to a flask containing compound **4** (20.0 mg, 0.063 mmol) at 0 °C. The reaction mixture was stirred at rt for 2 h, added with icy water (2 mL) and extracted with dichloromethane (5 mL × 2). The combined organic layers were dried over sodium sulfate, filtered and concentrated to give 4-methoxy-3-(7-methoxy-3-propyl-3*H*-[1,2,3]triazolo[4,5-d]pyrimidin-5-yl)benzene-1-sulfonyl chloride (25.0 mg) as a white solid.  ^1^H NMR (CDCl_3_, 300 MHz) δ 0.95 (t, *J* = 7.4 Hz, 3H), 1.97‒2.04 (m, 2H), 3.40 (s, 3H), 3.99 (s, 3H), 4.47 (t, *J* = 7.2 Hz, 2H), 7.25 (d, *J* = 4.5 Hz, 1H), 8.07 (d, *J* = 2.4 Hz, 1H), 8.20 (dd, *J* = 9.0 Hz, *J* = 2.6 Hz, 1H); ^13^C{^1^H} NMR (CDCl_3_, 75 MHz) δ 11.1, 23.0, 32.6, 48.7, 56.9 , 112.0, 125.4, 128.2, 129.1, 131.9, 136.9, 147.1, 155.8, 161.2. The above sulfonyl chloride was re-dissolved in dichloromethane (1 mL) and added to a solution of *N*-methylpiperazine (9 μL, 0.079 mmol), trimethylamine (18 μL, 0.13 mmol) and dichloromethane (2 mL). The reaction mixture was heated to refluxed for 4 h, cooled to rt, diluted with dichloromethane (5 mL), washed with water (3 mL) and saturated NaCl_(aq)_ (3 mL), dried over sodium sulfate, filtered and concentrated. The crude product was recrystallized (ethyl acetate/hexanes) to give **YL-9** as a white solid (21.0 mg, 0.046 mmol, 72%). Mp 116.0‒118.0 °C; ^1^H NMR (CDCl_3_, 300 MHz) δ 0.95 (t, *J* = 7.4 Hz, 3H), 1.94‒2.06 (m, 2H), 2.27 (s, 3H), 2.48 (t, *J* = 5.2 Hz, 4H), 3.07(t, *J* = 5.2 Hz, 4H), 3.38 (s, 3H), 3.90 (s, 3H), 4.46 (t, *J* = 7.3 Hz, 2H), 7.13(d, *J* = 8.8 Hz, 1H), 7.74(d, *J* = 2.3 Hz, 1H), 7.90 (dd, *J* = 8.8 Hz, *J* = 2.3 Hz, 1H); ^13^C{^1^H} NMR (CDCl_3_, 75 MHz) δ 11.1, 23.0, 32.6, 45.7, 45.9, 48.6, 53.9, 56.4, 111.4, 125.0, 128.2, 129.5, 132.2, 147.2, 155.9, 156.6, 159.5; HRMS (ESI) calcd for [M + H]^+^ (C_20_H_28_N_7_O_4_S) 462.1923, found 462.1923.

**Reference**

(1) Haning, H.; Niewöhner, U.; Schenke, T.; Lampe, T.; Hillisch, A.; Bischoff, E. *Bioorg. Med. Chem. Lett.* **2005**, *15*, 3900.
