# Supplementary figures and images for: Discovery of Novel Agents on Spindle Assembly Checkpoint to Sensitize Vinorelbine-Induced Mitotic Cell Death against Human Non-Small Cell Lung Cancers
Source: Int J Mol Sci. 2020 Aug 5;21(16):5608. doi: 10.3390/ijms21165608 (PMC7460560; doi:10.3390/ijms21165608)

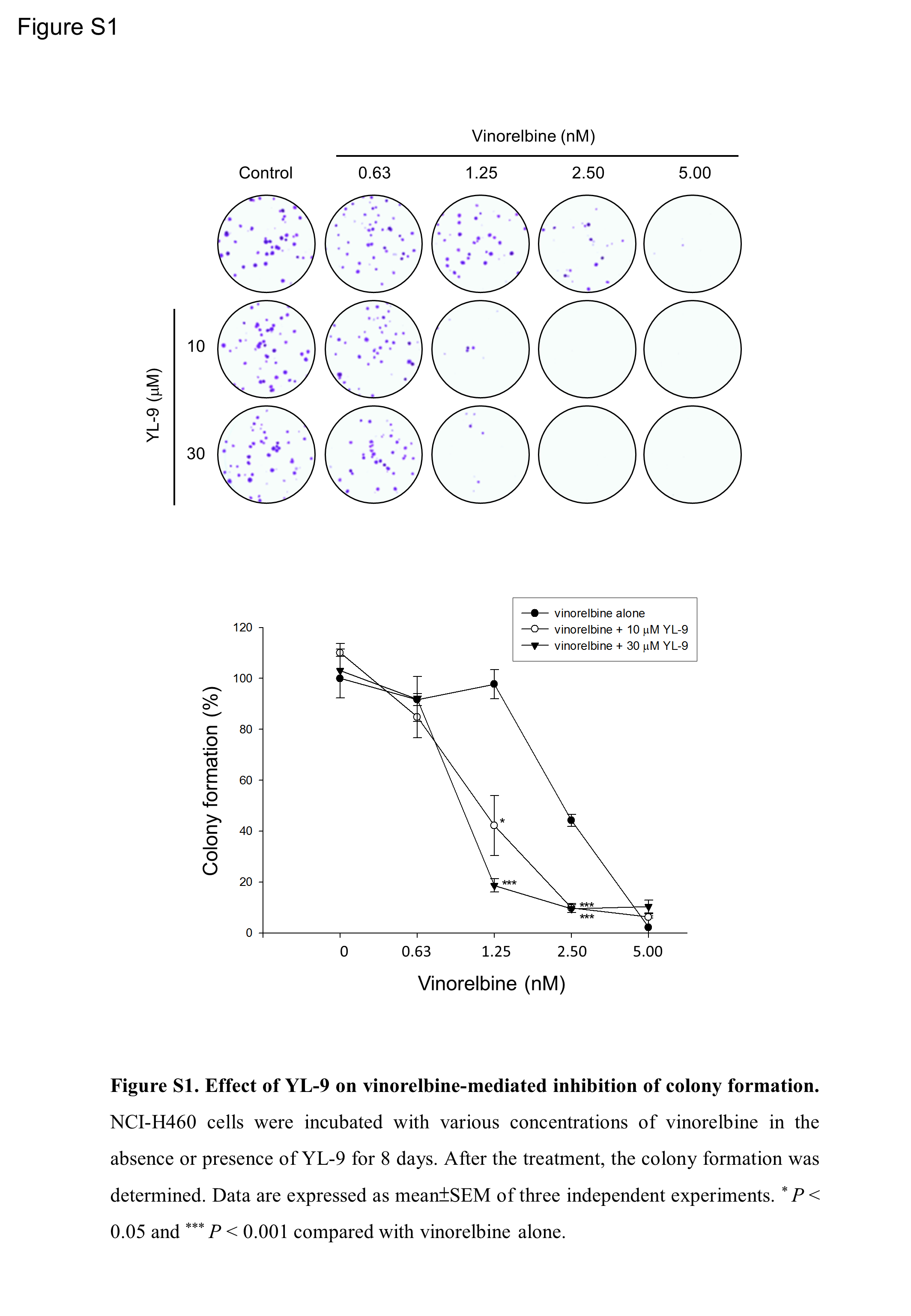

Supplement: Supplementary file 1 [file ijms-21-05608-s001.zip › Figure S1.tif]

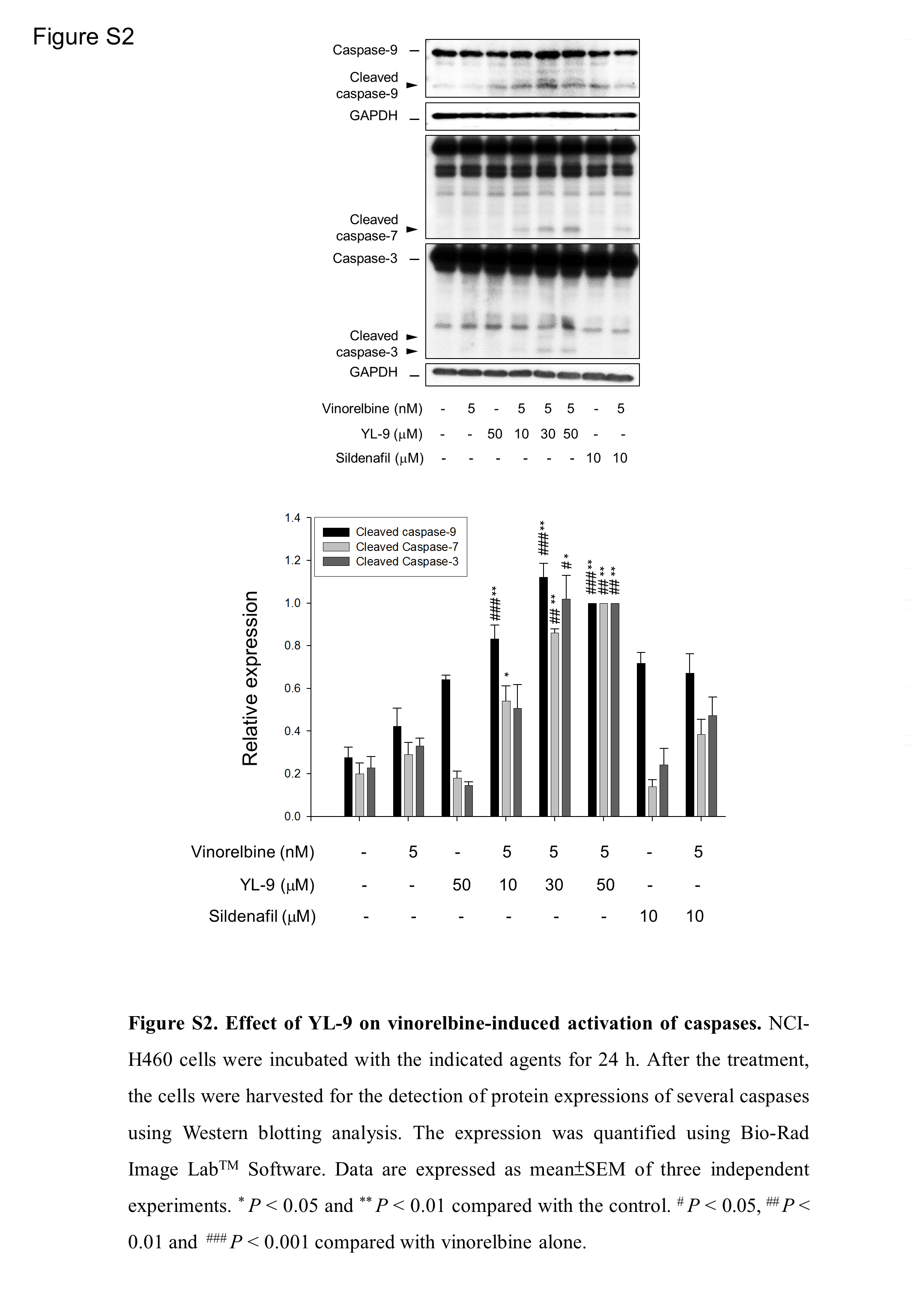

Supplement: Supplementary file 1 [file ijms-21-05608-s001.zip › Figure S2.tif]

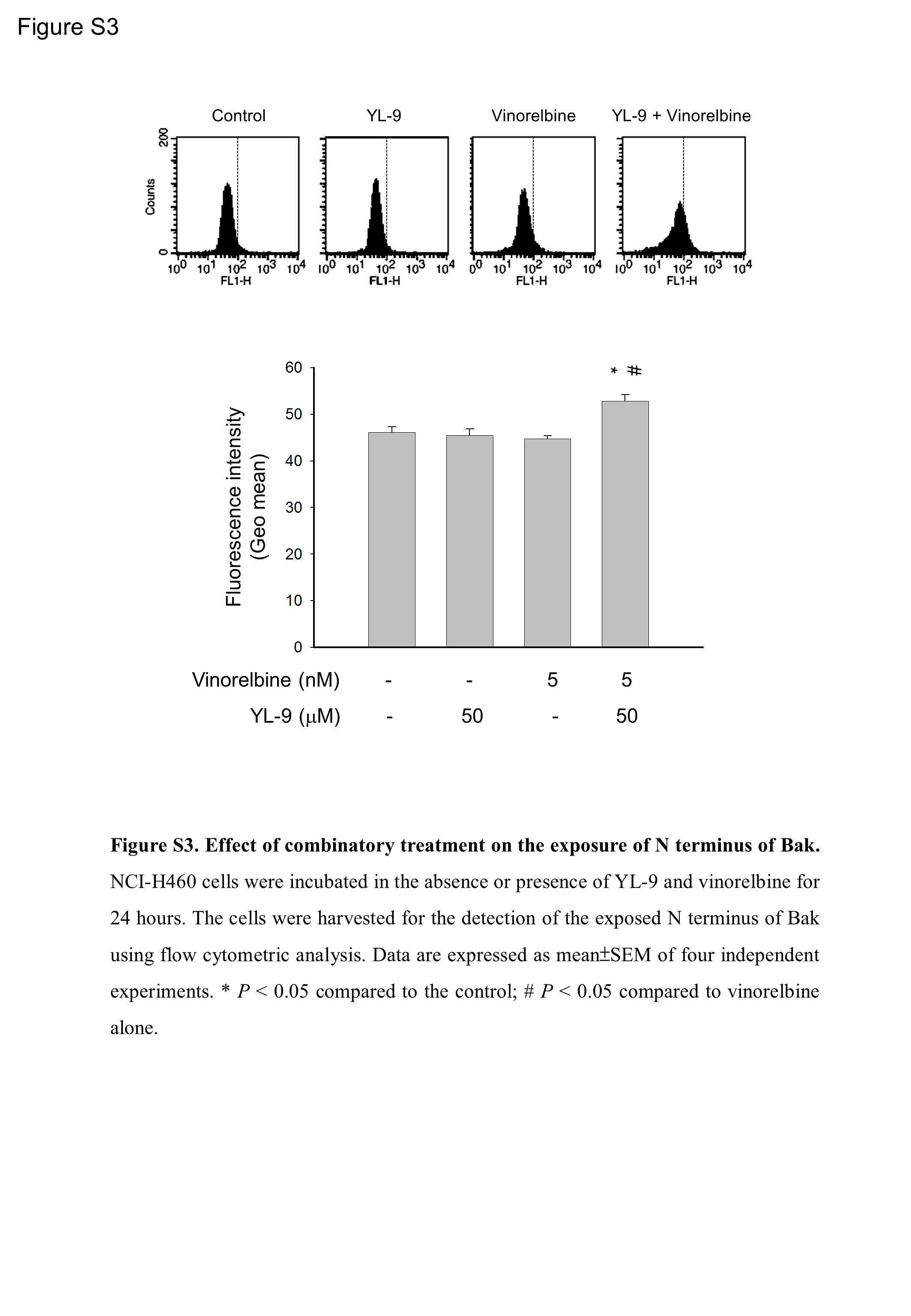

Supplement: Supplementary file 1 [file ijms-21-05608-s001.zip › Figure S3.tif]

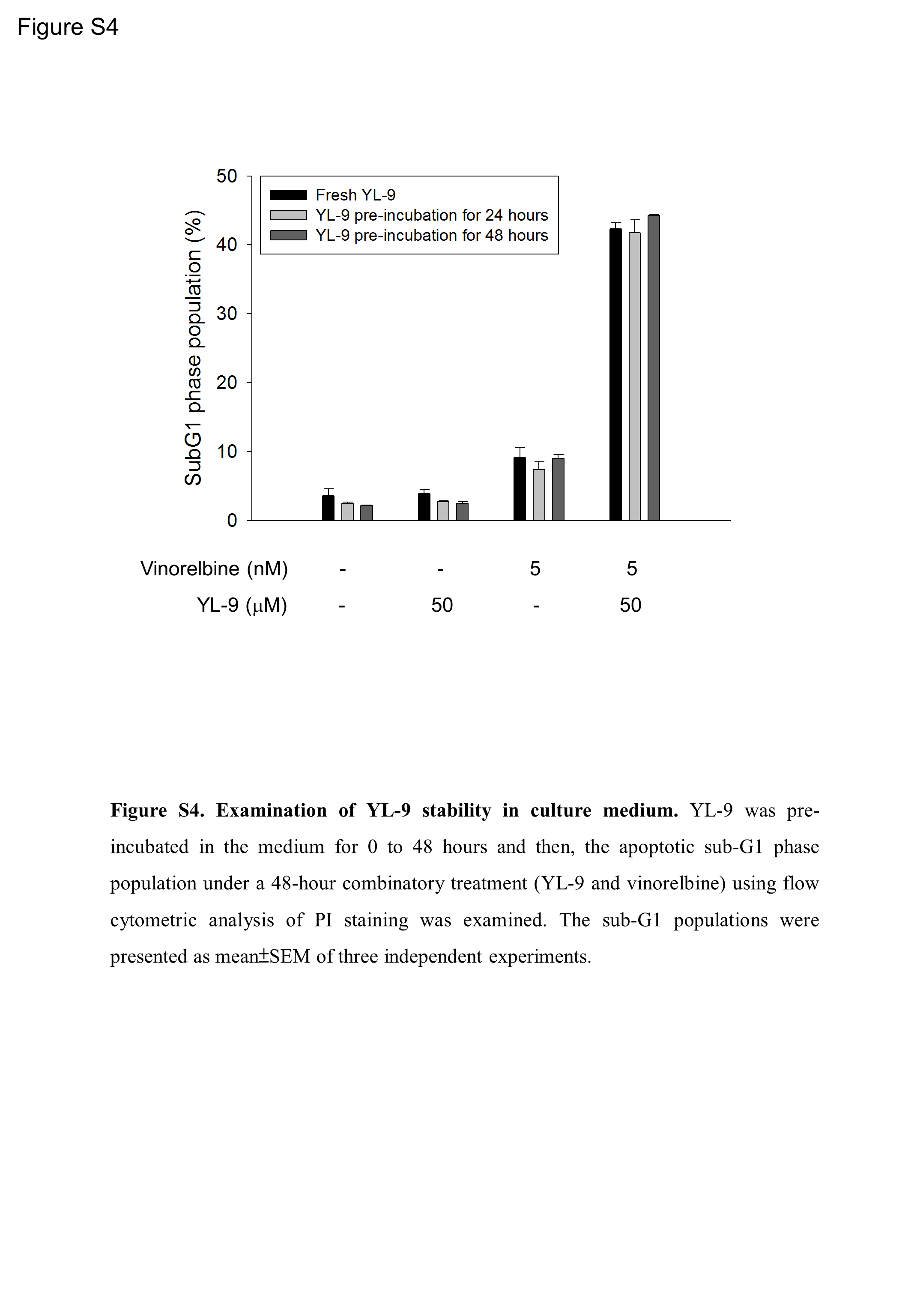

Supplement: Supplementary file 1 [file ijms-21-05608-s001.zip › Figure S4.tif]

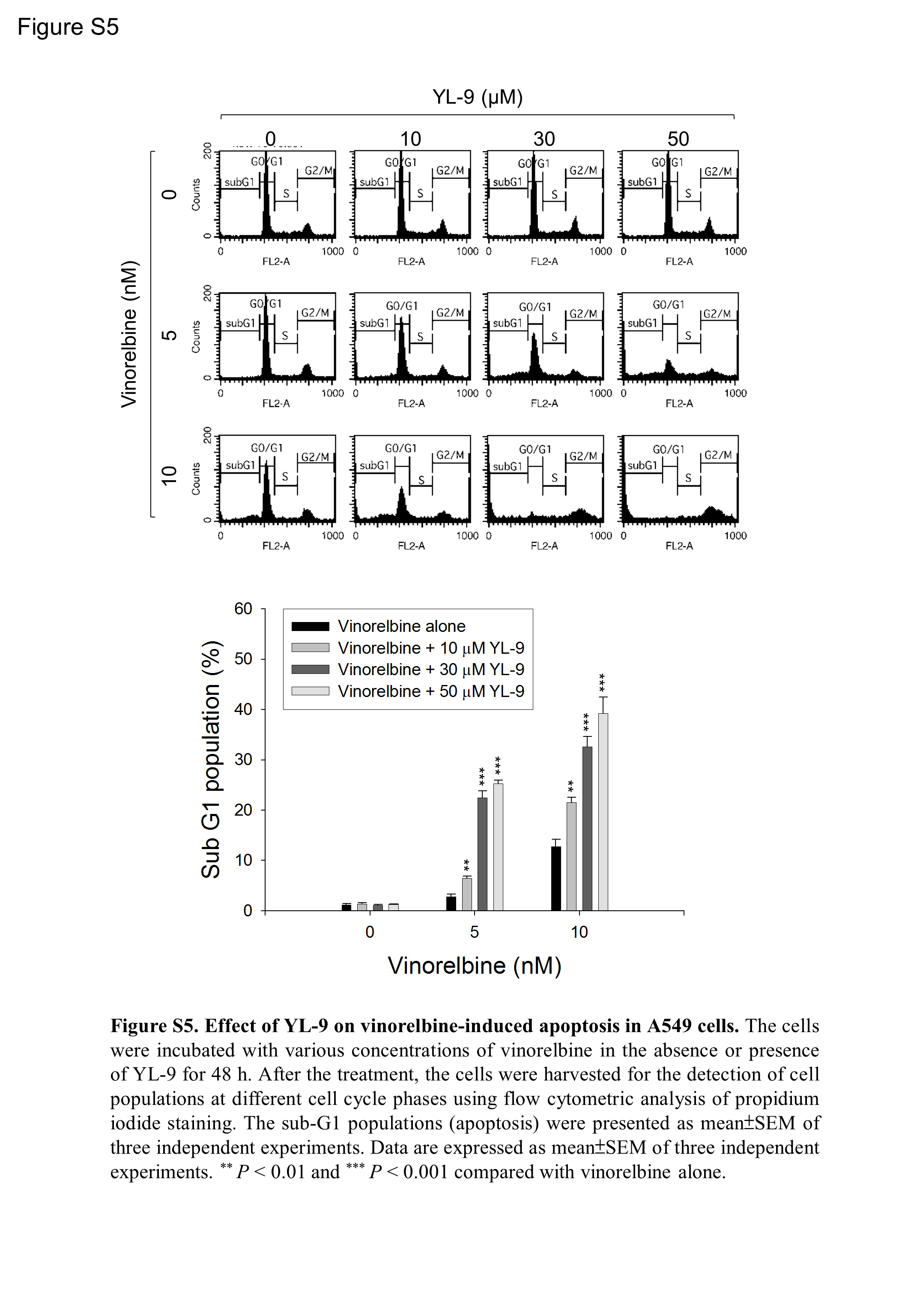

Supplement: Supplementary file 1 [file ijms-21-05608-s001.zip › Figure S5.tif]
